# Direct Lawsone O-Alkylation Employing Sulfonic Acid-Functionalized Chitosan as a Biodegradable Organocatalyst

**DOI:** 10.1021/acsomega.4c11019

**Published:** 2025-01-21

**Authors:** Iva S. de Jesus, Juliana Baptista de Pontes, Vania M. F. Paschoalin, Fernando de C. da Silva, Vitor Francisco Ferreira

**Affiliations:** † Department of Pharmaceutical Technology, Federal Fluminense University—UFF, Niteroi, Rio de Janeiro 24241-000, Brazil; ‡ Institute of Chemistry, Federal Fluminense University—UFF, Niteroi, Rio de Janeiro 24020-141, Brazil; § Department of Biochemistry, Institute of Chemistry, Federal University of Rio de Janeiro—UFRJ, Rio de Janeiro 21941-909, Brazil

## Abstract

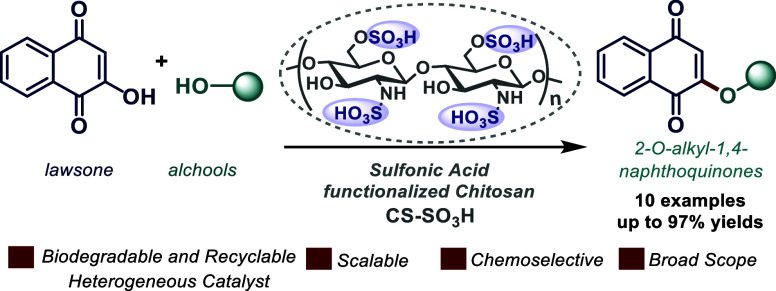

An environmentally friendly, cheap, and scalable protocol for direct lawsone O-alkylation employing different alcohols using acid chitosan (CS–SO_3_H) as a heterogeneous organocatalyst is described herein for the first time. A wide variety of alcohols can be converted with wide functional group tolerance and high atomic economy. Significantly, catalyst recycling and method chemoselectivity were also assessed.

## Introduction

The compound 1,4-naphthoquinone is a frequently encountered framework in numerous biologically active compounds.^1−6^ Its 2-alkoxy derivatives have demonstrated significant antiplatelet, antifungal, antiallergic, and anti-inflammatory activities.^7,8^ The extensive bioactivity profiles and drug-like properties of these compounds have attracted significant interest from synthetic organic chemists and pharmacologists, and significant efforts have been carried out to expand new sustainable methods for developing and modifying this important structure.

**Scheme 1:**
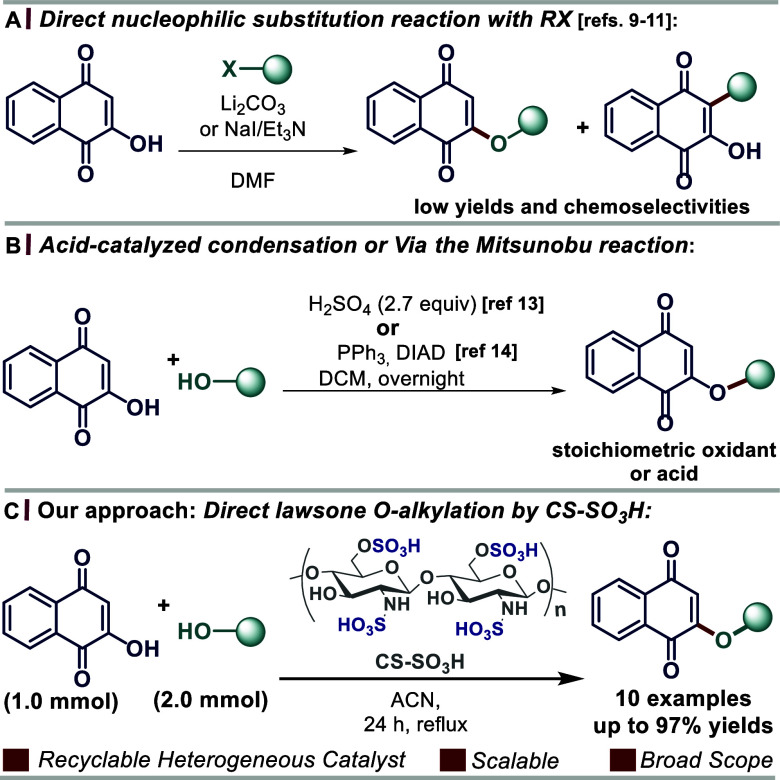
(a,b) Methodologies for the Synthesis of 2-*O*-alkyl 1,4-Naphthoquinone Derivatives (c) This Study: Direct Lawsone O-Alkylation with Different Alcohols Employing Acid Chitosan (CS–SO_3_H) as a Heterogeneous Organocatalyst

Direct lawsone C-2 hydroxy group SN_2_ alkylation by alkyl halides and alkyl sulfates in the presence of a stoichiometric amount of base encompasses one of the most traditional strategies for the synthesis of 2-*O*-alkyl-1,4-naphthoquinone derivatives (Scheme 1A).^9−11^ This condition provides a mixture of O- and C-alkylated products due to the tautomerization of 2-hydroxy-1,4-naphthoquinone (lawsone) in basic media.

Considering the minimization of hazardous waste and promotion of environmental sustainability, metal-free direct alcohol substitution emerges as a highly desirable approach. This method is significantly more eco-friendly and economical regarding steps and atoms, resulting in water as a byproduct.^12^ Reactions employing corresponding alcohols, however, have not been extensively studied to date.

Sabutskii and co-workers synthesized a series of 2-alkoxy-1,4-naphthoquinones by acid-catalyzed lawsone condensation employing different alcohols.^13^ The use of a stoichiometric amount of sulfuric acid in this case, however, represents a protocol drawback and limitation (Scheme 1B). Recently, Erasmus and co-workers reported lawsone 2-O-alkylation via the Mitsunobu reaction, although harsh reaction conditions limit its application (Scheme 1B).^14^

Despite the synthetic usefulness of the protocols, the development of an alternative approach for the chemoselective synthesis of 2-alkoxy-1,4-naphthoquinone derivatives under mild reaction conditions has not been carried out so far.

Heterogeneous catalysis field advances have expanded the synthetic availability for the preparation of densely functionalized molecules. In this sense, polymer-bound catalyst development and application efforts have increased throughout the past few years, due to their easy workup and product separation, as well as economic and environmental advantages.^15−18^ Chitosan (CS) is a good candidate in this context, as it is a natural, biocompatible, and biodegradable compound. The numerous basic groups present in this polysaccharide provide active chemical modification sites and the stabilization of homogeneous catalysts.^19−24^ Sulfonation represents one such chemical transformation, applied in the production of chitosan–SO_3_H (CS–SO_3_H), which has been employed as a solid acid catalyst in different organic reactions.^25−30^ In some cases, acid chitosan exhibits superior catalytic activity compared with the unmodified form. The enhanced catalytic activity of functionalized CS can be attributed to its increased surface area, higher availability of active sites on the catalyst surface, and improved thermal stability compared with pristine chitosan. These properties enable its effective application in high-temperature reactions.^31^

To the best of our knowledge, the CS-SO_3_H-catalyzed synthesis of 2-*O*-alkyl 1,4-naphthoquinone derivatives has not been reported to date. Thus, a convenient direct lawsone O-alkylation employing different alcohols using acid chitosan (CS–SO_3_H) as a biodegradable, accessible, and recyclable heterogeneous catalyst was employed herein, delivering a series of structurally and biorelevant 2-alkoxy-1,4-naphthoquinone derivatives (Scheme 1C).

## Results and Discussion

### Synthesis and Characterization of the CS–SO_3_H Catalyst

**Scheme 2:**
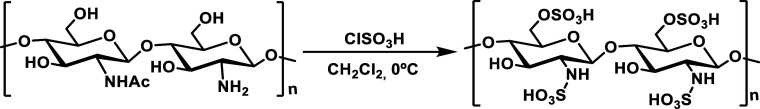
CS–SO_3_H Preparation

Chitosan–SO_3_H (CS–SO_3_H) was synthesized following a reported procedure.^29^ A solution of chlorosulfonic acid (2 mL) was added dropwise to a magnetically stirred suspension of chitosan (1.00 g) in dry dichloromethane (10 mL) at 0 °C over 1 h. Following the addition, the reaction mixture was stirred at room temperature for 2 h to ensure complete removal of HCl from the system. The resulting mixture was filtered and washed multiple times with methanol until a neutral pH was achieved. Finally, the product was dried at room temperature, yielding chitosan–SO_3_H as a white solid (Scheme 2).

**Figure 1:**
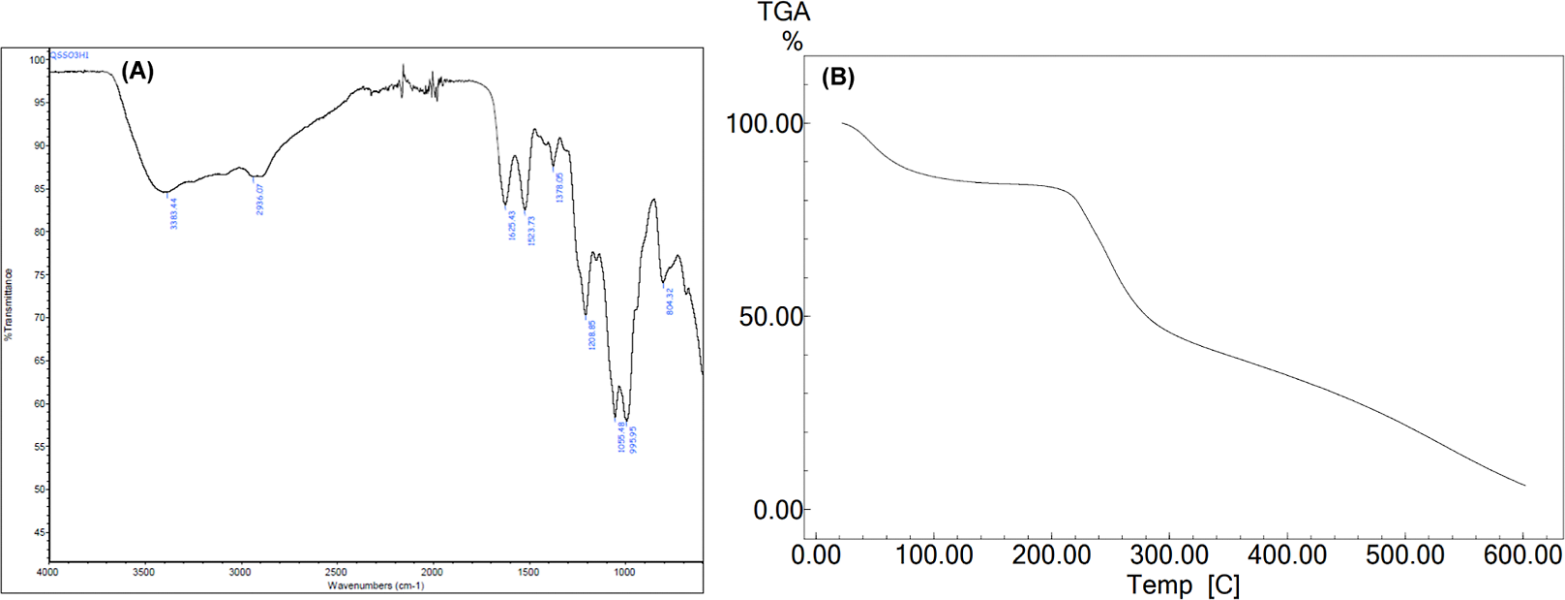
(a) FT-IR spectrum of CS–SO_3_H. (b) TGA curve of CS–SO_3_H.

The synthesized CS–SO_3_H was comprehensively characterized using various analytical techniques including Fourier transform infrared spectroscopy (FTIR), thermogravimetric analysis (TGA), field emission scanning electron microscopy (FE-SEM), and energy dispersive X-ray spectroscopy (EDS).^29^ The FTIR spectrum confirmed the presence of distinct functional groups in CS–SO_3_H. Based on prior studies and the current data, characteristic bands observed at 1208 and 1055 cm^–1^ correspond to the S=O stretching vibrations of the –SO_3_H group in −O–SO_3_H and NH–SO_3_H, respectively. Additionally, the peak at 804 cm^–1^ is attributed to the stretching vibration of the S–N bond in –HN–SO_3_H (Figure 1a). The thermal stability of the prepared catalyst (CS–SO_3_H) was evaluated through TGA within the temperature range of 50–500 °C. The first weight loss (approximately 5%) at 90 °C is due to the removal of solvent and other small molecules. The degradation of chitosan polysaccharide and SO_3_H groups is the second largest loss of approximately 20–75% in the 250–300 °C range (Figure 1b).

**Figure 2:**
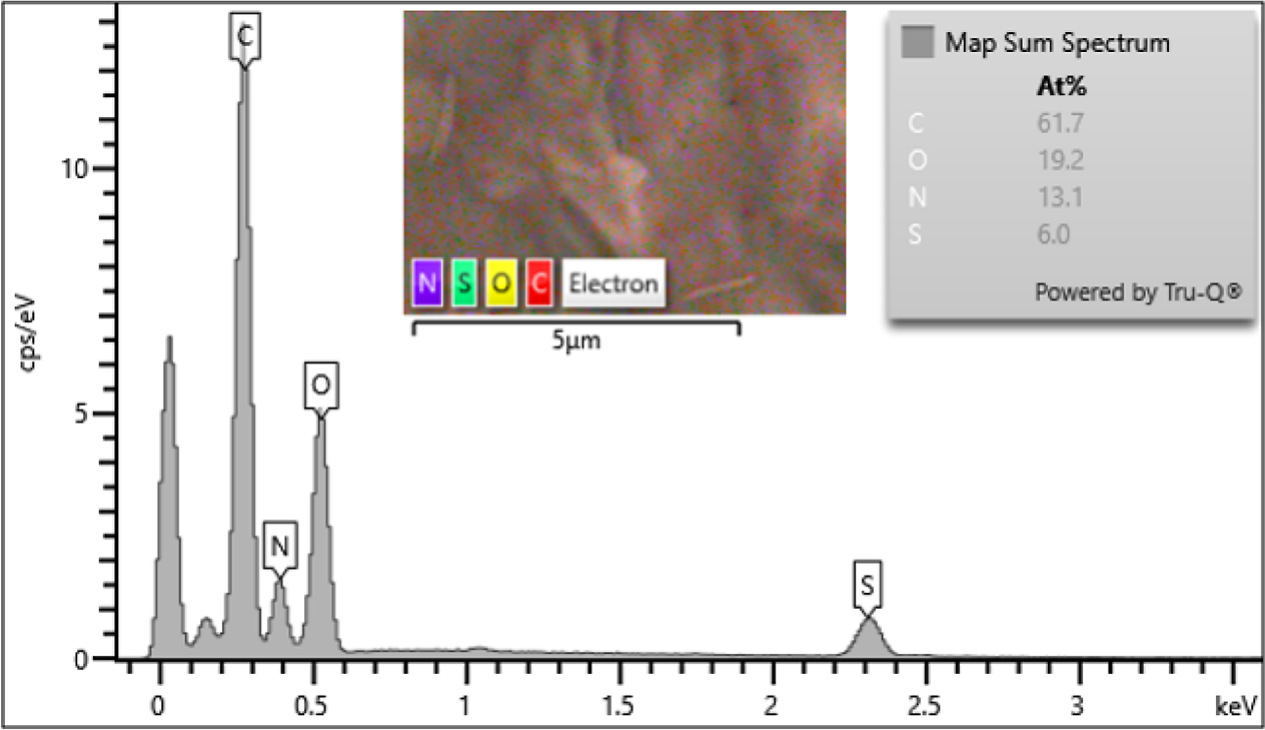
EDS spectra of CS–SO_3_H.

**Figure 3:**
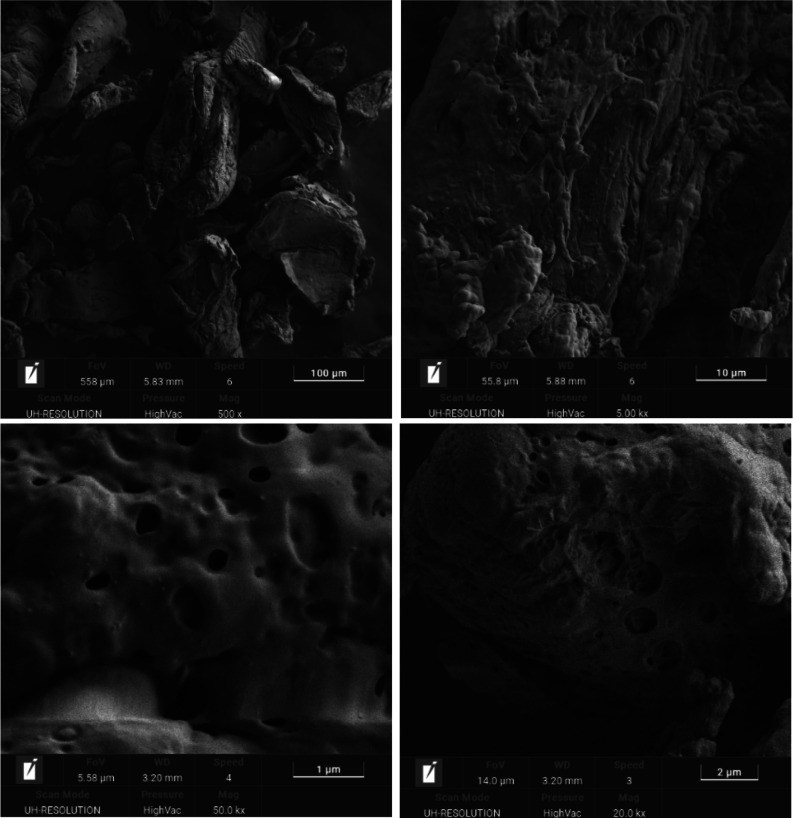
FE-SEM images of CS–SO_3_H.

EDS analysis determined the elemental composition of the material, confirming the presence of carbon, oxygen, nitrogen, and sulfur with weight percentages of 61.7%, 19.2%, 13.1%, and 6.0%, respectively. These findings confirmed the successful incorporation of −SO_3_H groups on the chitosan backbone (Figure 2). In addition, the surface morphology, particle characteristics, and size distribution of CS–SO_3_H were analyzed by using FE-SEM (Figure 3). The findings revealed a uniform fibrous surface featuring voids and cracks, which acted as active sites for the specific reaction.

### Evaluation of Catalytic Activity of CS–SO_3_H for Direct Lawsone O-Alkylation

Our study began through the reaction between lawsone **1** and ethyl alcohol **2a** as model substrates for the evaluation of the effect of various parameters on the reaction outcome. Following preliminary experiments (Supporting Information for details), 100 mg of the catalyst, 1.0 equiv of **1**, and 2.0 equiv of **2a** in acetonitrile were noted as adequate for the preparation of the desired product (**3a**), with an 86% yield, after 24 h under reflux (Table 1, entry 1). Entries 2–12 summarize some deviations from optimized conditions. According to Table 1, when 1.5 equiv of **2a** was used, the yield decreased from 86% to 41%, which is a reduction by half (entry 2). Solvent studies indicate that acetonitrile is the most efficient solvent to promote the desired transformations (Table 1, entries 3–6). When conducting the reaction in the presence of *p*-toluene sulfonic acid (*p*-TSA) as the catalyst, product **3a** was obtained at a very low yield (Table 1, entry 7). Another advantage of CS–SO_3_H compared to traditional *p*-TSA encompasses a simple and clean catalyst separation from the reaction mixture and the possibility of recycling. The reaction mixture was then tested in the presence of pristine chitosan (low molecular weight), but the desired product was not formed (Table 1, entry 8). The presence of multiple SO_3_H groups in the CS–SO_3_H structure seems to play an important role in the formation of the desired product due to lawsone activation and corresponding intermediate stabilization. The use of a lower amount of CS–SO_3_H was detrimental to the reaction, resulting in a significant decrease in the chemical yield of **3a** (Table 1, entries 9 and 10). As a control experiment, the reaction was performed in the absence of a catalyst and a heating source (Table 1, entries 11 and 12, respectively). The desired product, however, was not formed in either case.

**Table 1:**
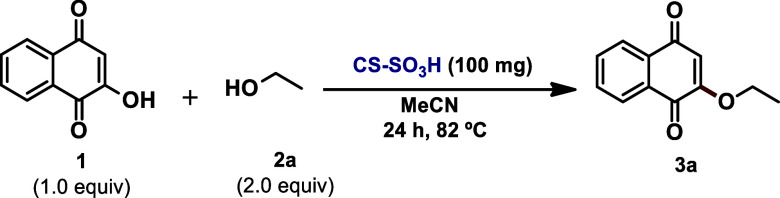
Optimization and Control Studies^a^^,^^b^

entry	deviation from optimum conditions	yields **3a** (%)^c^
1	MeCN	86
2	1.5 equiv of **2a**	41
3	THF	47
4	DMSO	25
5	acetone	28
6	dichloroethane	39
7	*p*-TSA	51
8	chitosan	nr
9	CS–SO_3_H (10 mg)	nr
10	CS–SO_3_H (50 mg)	trace
11	without catalyst	nr
12	room temperature	nr

a
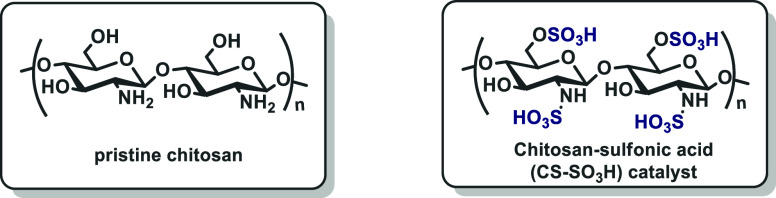

b Reaction conditions: **1** (1.0 mmol), **2a** (2.0 mmol), and catalyst (100 mg) in MeCN (10.0 mL).

c Isolated yield.

**Scheme 3:**
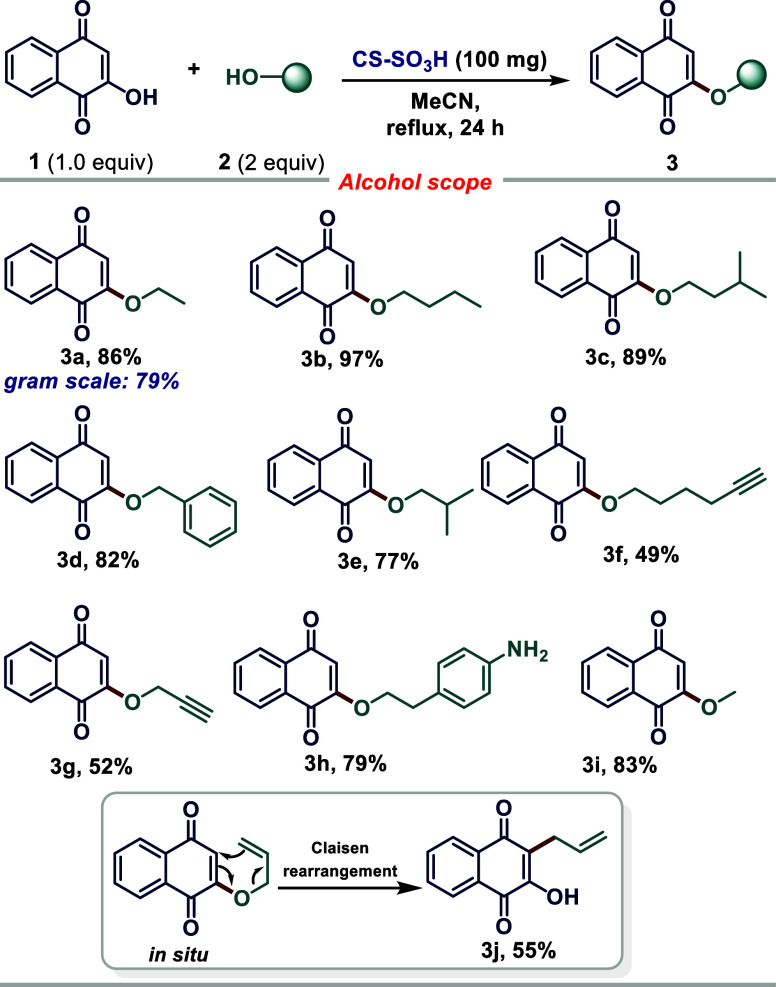
Scope Study of 2-Alkoxy-1,4-naphthoquinone Synthesis Employing CS–SO_3_H as a Biodegradable Catalyst

Following optimal condition establishment, the scope and limitations of the newly developed approach were assessed. Method generality was evaluated employing a wide variety of alcohols (Scheme 3). The results indicate good tolerance to different functional groups, resulting in satisfactory corresponding 2-alkoxy-1,4-naphthoquinone derivatives **3a–3j** with good yields. Highly functionalized primary alkyl alcohols were compatible with this protocol and resulted in good yields. The alcohol containing an NH_2_ moiety at the aryl moiety was evaluated (**3h**), resulting in a 79% yield. Products containing a terminal alkyne (**3f** and **3g**) can also be functionalized through 1,3-dipolar reactions. High method chemoselectivity was noted, as only the *O*-alkylated product was obtained. The C3-alkylated product **3j** is assumed as obtained via Claisen rearrangement.

To demonstrate the method’s robustness and efficiency, the standard reaction using model substrate **1a** was conducted on a 5.0 mmol scale. The desired product **3a** was obtained in 79% yield with no loss of reactivity or selectivity, emphasizing this protocol’s potential as an environmentally friendly methodology.

**Scheme 4:**
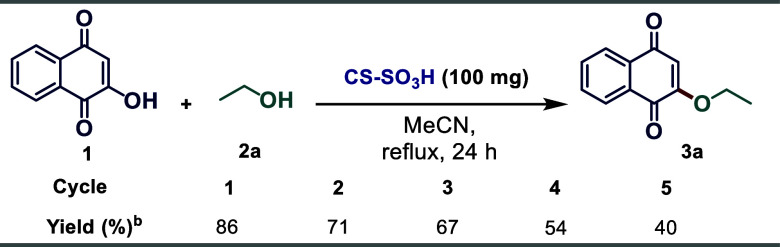
Evaluation of Catalyst Recycling

The recyclability of CS–SO_3_H was further assessed, as the ability to repeatedly use a heterogeneous catalyst is a critical factor for its practical applications. After separation from the reaction mixture by filtration, the catalyst was washed with acetonitrile and dried in the air (Scheme 4). The catalyst was then reused in the model reaction for five consecutive cycles. A gradual decrease in product yield was observed with each reuse: (run 1, 86%; run 2, 71%; run 3, 67%; run 4, 54%; and run 5, 40%). We assume that the decrease in the catalytic activity of CS–SO_3_H may be associated with the potential loss of sulfonic groups in each cycle.

**Scheme 5:**
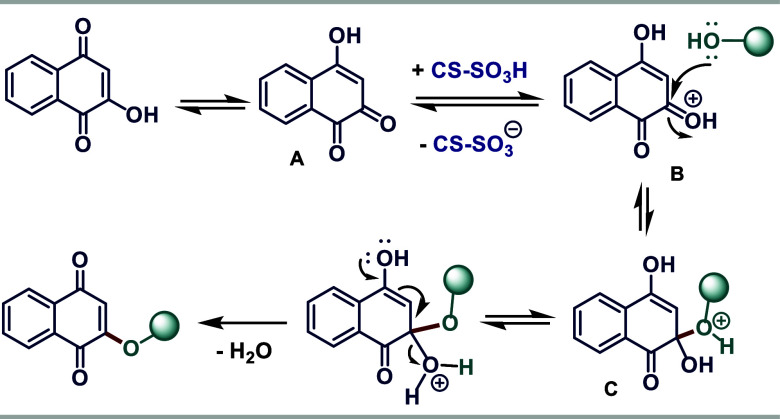
Mechanism Proposal for the Synthesis of 2-Alkoxy-1,4-naphthoquinones Employing CS–SO_3_H as a Biodegradable Catalyst

Based on the results and previous reports,^13^ a plausible reaction mechanism is proposed, indicating the catalytic activation of CS-SO_3_H (Scheme 5). In the first step, tautomeric 1,2-naphthoquinone **A** is formed. The protonation of the carbonyl group by CS–SO_3_H results in species **B**, which undergoes a nucleophilic OH group attack on the alcohol to an activated carbonyl group, leading to the formation of a tetrahedral intermediate **C**. Finally, this species undergoes dehydration, resulting in the desired 2-alkoxy-1,4-naphthoquinone.

**Scheme 6:**
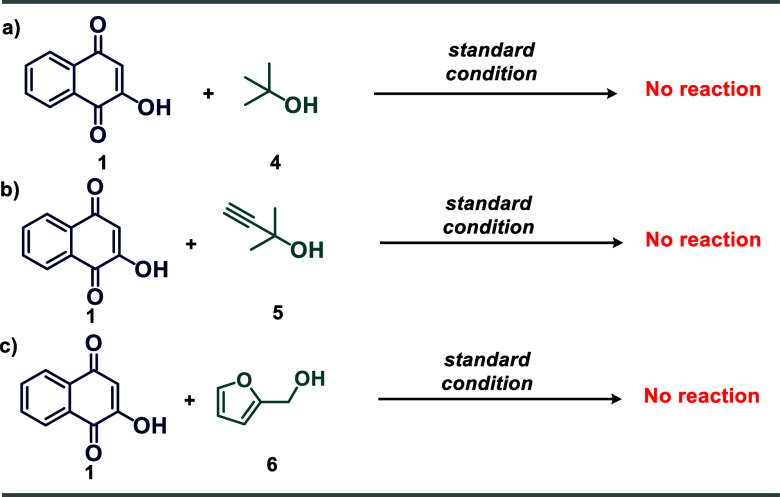
Control Experiments

Some control reactions, shown in Scheme 6, were undertaken to support the mechanical proposal described in Scheme 4. Under the QS-SO_3_H-promoted condition, compounds **4**–**5** did not react with lawsone, with reagents being recovered (equations A and B of Scheme 6). In this way, these results suggest that tautomeric 1,2-naphthoquinone **A** should be involved in the reaction of O-alkylation, which is activated by CS–SO_3_H, resulting in the electrophilic species **B**. Thus, tertiary alcohols with low nucleophilicity do not react with lawsone under these conditions. The reaction between lawsone and furfuryl alcohol **6** also did not provide the product of interest. However, we assume that the presence of sulfonic groups in the catalyst led to the degradation of **6**.

## Conclusions

A series of 2-alkoxy-1,4-naphthoquinones were synthesized herein by employing an environmentally benign, cheap, and scalable protocol via direct lawsone O-alkylation, employing different alcohols using acid chitosan (CS–SO_3_H) as a heterogeneous organocatalyst. The reaction was highly selective, displaying high atomic economy and employing a cheap, broad variety of alcohols with a wide functional group tolerance and a recycling and renewable catalyst. This methodology provides a useful route to produce 2-alkoxy-1,4-naphthoquinones, which can be applied in constructing molecules with interesting biological properties and pharmaceutical potential.

## Experimental Section

### General Experimental Information

All chemicals were purchased and used without further purification. Anhydrous solvents were either purchased or dried employing standard drying agents and freshly distilled before use. Reactions were monitored by thin-layer chromatography (TLC) (Silica gel 60 F254, Merck KGaA, Darmstadt, Germany). Flash column chromatography was performed using Silica Gel 60 M (40–63 μm, Machery Nagel GmbH & Co., Düren, Germany). Melting points were obtained on a Fisatom 430D apparatus and uncorrected. Infrared spectra were recorded on an FT-IR Thermo Nicolet IS-50 equipment operated in the ATR mode (32 scans) (resolution = 4 cm^–1^). ^1^H and ^13^C NMR spectra were acquired on a Bruker Advance NEO spectrometer operating at 500 MHz, employing a direct broadband probe at 125 MHz in CDCl_3_ or DMSO-*d*_6_ at 25 °C. Chemical shifts (δ) are reported in parts per million relative to residual solvent signals, and coupling constants (*J*) are reported in hertz. Multiplicities are described as brs = broad signal, s = singlet, d = doublet, t = triplet, q = quartet, dd = doublet of doublets, dt = doublet of triplets, and m = multiplet. APPI-Q-TOFMS measurements were obtained on a mass spectrometer equipped with an automatic syringe pump for sample injection.

Chitosan–SO_3_H (CS–SO_3_H) was synthesized following a literature process^24^ (characterization data and FT-IR spectra are provided in the Supporting Information).

### General Procedure: 2-Alkoxy-1,4-naphthoquinone Synthesis

#### General Procedure on a 1.0 mmol Scale (**3a–j**)

Lawsone (1.0 mmol, 1.0 equiv), the appropriate alcohol (2.0 mmol, 2.0 equiv), and 0.100 g of chitosan–SO_3_H were mixed in a 25 mL round-bottom flask containing a magnetic stir bar. The mixture was then dissolved in 10.0 mL of acetonitrile and refluxed at 82 °C. TLC monitored reagent consumption. After 24 h of reaction, product formation was observed, and the reaction was terminated. The reaction mixture was then cooled to room temperature, the crude material was filtered to recover the catalyst, the residual solvent was evaporated under reduced pressure, and the solid catalyst was fully recovered. The product was purified by flash column chromatography (within the eluent indicated in each case), providing the respective product **3**. 2-Alkoxy-1,4-naphthoquinones **3a–l** were determined by ^1^H and ^13^C NMR spectroscopy (characterization data and ^1^H and ^13^C NMR spectra are provided in the Supporting Information).

### General Procedure on a 5 mmol Scale

The aforementioned protocol was followed using lawsone (870 mg, 5 mmol), ethyl alcohol **2a** as the solvent (10.0 mL), and chitosan–SO_3_H (500 mg). Flash chromatography was carried out on silica (0–30% EtOAc in hexanes), resulting in product **3a** (797 mg, 79% yield), which is a light brown solid.

#### 2-Ethoxynaphthalene-1,4-dione (**3a**)^32^

(173 mg, 86% yield), light brown solid, purified by flash column chromatography (30% EtOAc in hexanes). mp: 115–118 °C. ^1^H NMR (500 MHz, CDCl_3_): δ 8.13–8.01 (m, 2H), 7.75–7.64 (m, 2H), 6.13 (s, 1H), 4.08 (q, *J* = 7.0 Hz, 2H), 1.51 (t, *J* = 7.0 Hz, 3H). ^13^C NMR (126 MHz, CDCl_3_): δ 185.02, 180.18, 159.69, 134.23, 133.27, 131.99, 131.13, 126.68, 126.10, 110.20, 65.34, 13.93. **IR** (ATR, *v*_max_/cm^–1^): 3073, 2987, 1678, 1654, 1608, 1242, 1206, 1044, 817. HRMS (ESI): *m*/*z* calcd for C_12_H_10_NaO_3_ [M + Na]^+^ 225.0528; found, 225.0514.

#### 2-Butoxynaphthalene-1,4-dione (**3b**)

(223 mg, 97% yield), orange solid, purified by flash column chromatography (30% EtOAc in hexanes). mp: 108–109 °C. ^1^H NMR (500 MHz, CDCl_3_): δ 8.13–8.02 (m, 2H), 7.75–7.65 (m, 2H), 6.14 (s, 1H), 4.00 (t, *J* = 6.6 Hz, 2H), 1.94–1.80 (m, 2H), 1.58–1.43 (m, 2H), 0.98 (t, *J* = 7.4 Hz, 3H). ^13^C NMR (126 MHz, CDCl_3_): δ 185.06, 180.15, 159.91, 134.20, 133.24, 132.02, 131.18, 126.66, 126.09, 110.18, 69.38, 30.24, 19.12, 13.70. FT-IR (ATR, *v*_max_/cm^–1^): 3054, 2955, 1681, 1605, 1362, 1245, 1042, 738. HRMS (ESI): *m*/*z* calcd for C_14_H_14_NaO_3_ [M + Na]^+^ 253.0841; found, 253.0830.

#### 2-(Isopentyloxy)naphthalene-1,4-dione (**3c**)

(217 mg, 89% yield), yellow solid, purified by flash column chromatography (30% EtOAc in hexanes). mp: 71–72 °C. ^1^H NMR (500 MHz, CDCl_3_): δ 8.13–8.03 (m, 2H), 7.75–7.64 (m, 2H), 6.13 (s, 1H), 3.83 (d, *J* = 6.1 Hz, 1H), 3.77 (d, *J* = 6.7 Hz, 1H), 2.08–1.94 (m, 1H), 1.65–1.49 (m, 1H), 1.30 (dt, *J* = 13.6, 7.6 Hz, 1H), 1.07–0.92 (m, 6H). ^13^C NMR (126 MHz, CDCl_3_): δ 185.08, 180.08, 160.03, 134.20, 134.17, 133.25, 133.23, 132.04, 131.22, 110.15, 74.24, 33.98, 26.01, 16.37, 11.19. FT-IR (ATR, *v*_max_/cm^–1^): 3057, 2958, 1680, 1650, 1247, 1038, 725. HRMS (ESI): *m*/*z* calcd for C_15_H_16_NaO_3_ [M + Na]^+^ 267.0997; found, 267.0983.

#### 2-(Benzyloxy)naphthalene-1,4-dione (**3d**)

(216 mg, 82% yield), yellow solid, purified by flash column chromatography (7% EtOAc in hexanes). mp: 148–149 °C. ^1^H NMR (500 MHz, CDCl_3_): δ 8.17–8.01 (m, 2H), 7.75–7.65 (m, 2H), 7.48–7.31 (m, 5H), 6.22 (s, 1H), 5.12 (s, 2H). ^13^C NMR (126 MHz, CDCl_3_): δ 184.91, 180.03, 159.31, 134.27, 134.18, 133.35, 131.96, 131.16, 128.90, 128.77, 127.63, 126.69, 126.15, 111.20, 71.12. FT-IR (ATR, *v*_max_/cm^–1^): 3053, 1681, 1651, 1247, 1011, 747. HRMS (ESI): *m*/*z* calcd for C_17_H_12_NaO_3_ [M + Na]^+^ 287.0684; found, 287.0687.

#### 2-Isobutoxynaphthalene-1,4-dione (**3e**)

(177 mg, 77% yield), light brown solid, purified by flash column chromatography (10% EtOAc in hexanes). mp: 84–88 °C. ^1^H NMR (500 MHz, CDCl_3_): δ 8.13–8.03 (m, 2H), 7.76–7.65 (m, 2H), 6.13 (s, 1H), 3.75 (d, *J* = 6.7 Hz, 2H), 2.26–2.18 (m, 1H), 1.05 (d, *J* = 6.8 Hz, 6H). ^13^C NMR (126 MHz, CDCl_3_): δ 185.07, 180.08, 159.96, 134.17, 133.24, 132.03, 131.21, 126.63, 126.09, 110.18, 75.59, 27.64, 19.11. FT-IR (ATR, *v*_max_/cm^–1^): 3055, 2960, 1652, 1596, 1266, 1038, 721. HRMS (ESI): *m*/*z* calcd for C_14_H_14_NaO_3_ [M + Na]^+^ 253.0841; found, 253.0848.

#### 2-(Hex-5-yn-1-yloxy)naphthalene-1,4-dione (**3f**)

(124 mg, 49% yield), yellow solid, purified by flash column chromatography (10% EtOAc in hexanes). mp: 89–91 °C. ^1^H NMR (500 MHz, CDCl_3_): δ 8.16–7.99 (m, 2H), 7.79–7.62 (m, 2H), 6.14 (s, 1H), 4.03 (t, *J* = 6.4 Hz, 2H), 2.30–2.26 (m, 2H), 2.07–1.95 (m, 3H), 1.75–1.70 (m, 2H). ^13^C NMR (126 MHz, CDCl_3_): δ 184.99, 180.04, 159.75, 134.24, 133.29, 131.99, 131.15, 126.66, 126.11, 110.25, 83.62, 69.07, 68.97, 27.29, 24.76, 18.06. FT-IR (ATR, *v*_max_/cm^–1^): 3286, 3244, 2956, 1631, 1605, 1248, 1026, 698. HRMS (ESI): *m*/*z* calcd for C_16_H_14_NaO_3_ [M + Na]^+^ 277.0841; found, 277.0839.

#### 2-(Prop-2-yn-1-yloxy)naphthalene-1,4-dione (**3g**)^32^

(110 mg, 52% yield), light yellow solid, purified by flash column chromatography (DCM as an eluent). mp: 146–149 °C. ^1^H NMR (500 MHz, CDCl_3_): δ 8.19–8.01 (m, 2H), 7.77–7.68 (m, 2H), 6.34 (s, 1H), 4.79 (d, *J* = 2.4 Hz, 2H), 2.65 (s, 1H). ^13^C NMR (126 MHz, CDCl_3_): δ 183.90, 178.77, 158.00, 141.59, 138.17, 133.32, 132.41, 128.28, 127.40, 110.34, 96.43, 73.72, 68.29. FT-IR (ATR, *v*_max_/cm^–1^): 3250, 3053, 1680, 1603, 1261, 1244, 1040, 737.

#### 2-(4-Aminophenethoxy)naphthalene-1,4-dione (**3h**)

(247 mg, 61% yield), red solid, purified by flash column chromatography (10–30% EtOAc in hexanes). mp: 107–110 °C. ^1^H NMR (500 MHz, CDCl_3_): δ 8.13–8.07 (m, 2H), 8.05 (s, 1H), 7.75 (tt, *J* = 7.5, 1.1 Hz, 1H), 7.65 (tt, *J* = 7.5, 1.1 Hz, 1H), 7.55 (s, 1H), 7.30–7.19 (m, 4H), 6.37 (d, *J* = 0.9 Hz, 1H), 4.39 (t, *J* = 6.9 Hz, 2H), 2.98 (t, *J* = 6.9 Hz, 2H). ^13^C NMR (126 MHz, CDCl_3_): δ 182.85, 181.01, 159.88, 143.75, 135.08, 133.96, 133.90, 132.20, 131.33, 129.34, 129.12, 125.50, 125.14, 121.81, 102.33, 63.07, 33.37. FT-IR (ATR, *v*_max_/cm^–1^): 3272, 2945, 1714, 1681, 1594, 1514, 1177, 727.

#### 2-Methoxynaphthalene-1,4-dione (**3i**)^32^

(156 mg, 83% yield), light yellow solid, purified by recrystallization in ethanol mp: 167–170 °C. ^1^H NMR (500 MHz, CDCl_3_): δ 8.14–8.01 (m, 2H), 7.78–7.63 (m, 2H), 6.16 (s, 1H), 3.89 (s, 3H). ^13^C NMR (126 MHz, CDCl_3_): δ 184.85, 180.11, 160.44, 134.35, 133.36, 132.02, 131.05, 126.71, 126.19, 109.90, 56.44. FT-IR (ATR, *v*_max_/cm^–1^): 3048, 1679, 1602, 1241, 1043, 722.

#### 2-Allyl-3-hydroxynaphthalene-1,4-dione (**3j**)^32^

(117 mg, 55% yield), light brown oil, purified by flash column chromatography (30% EtOAc in hexanes). ^1^H NMR (CDCl_3_, 500 MHz): δ 8.18–8.03 (m, 2H), 7.76 (td, *J* = 7.6, 1.4 Hz, 1H), 7.69 (td, *J* = 7.5, 1.3 Hz, 1H), 7.36 (s, 1H), 5.97–5.86 (m, 1H), 5.17 (dd, *J* = 17.1, 1.6 Hz, 1H), 5.05 (dd, *J* = 10.0, 1.6 Hz, 1H), 3.37 (d, *J* = 6.5 Hz, 2H).

## Data Availability

The data presented in this study are available in the published article and its online Supporting Information.

## Supporting Information Available

Details of the complete experimental procedures, characterization data, nuclear magnetic resonance spectra, and high-resolution mass spectrometry (PDF)
