# Supplementary material for: Direct Lawsone O-Alkylation Employing Sulfonic Acid-Functionalized Chitosan as a Biodegradable Organocatalyst
Source: ACS Omega. 2025 Jan 21;10(4):4163–9. doi: 10.1021/acsomega.4c11019 (PMC11800155; doi:10.1021/acsomega.4c11019)
Supplement: Supplementary file 1 — ao4c11019_si_001.pdf [file ao4c11019_si_001.pdf]

Supporting Information

# Direct Lawsone *O*-alkylation Employing Sulfonic Acid-functionalized Chitosan as a Biodegradable Organocatalyst

Iva S. de Jesus,*[a,b] Juliana Baptista de Pontes,[a,b] Vania M. F. Paschoalin,[c] Fernando de C. da Silva,[b] and Vitor Francisco Ferreira*[a]

[a] *Department of Pharmaceutical Technology, Federal Fluminense University – UFF, Niteroi, RJ, 24241-000, Brazil.*

[b] *Institute of Chemistry, Federal Fluminense University – UFF, Niteroi, RJ, 24020-141, Brazil*

[c] *Department of Biochemistry, Institute of Chemistry, Federal University of Rio de Janeiro – UFRJ -, Rio de Janeiro, RJ, 21941-909, Brazil.*

Corresponding authors: * *E-mail: ivasouza.quimica@gmail.com; vitorferreira@id.uff.br*

1. **General Considerations** ........................................ 3
2. **Synthesis and characterization of Chitosan-$SO_3H$** ........................................ 3
3. **Scale-up Experiment** ........................................ 7
4. **General Procedure for O-alkylation of Lawsone** ........................................ 8
5. **Compound Characterization Data** ........................................ 8
6. **$^{1}H$, $^{13}C$ NMR and HRMS spectra** ........................................ 12

## 1. General Considerations

All chemicals were purchased and used without further purification. Anhydrous solvents were either purchased or dried employing standard drying agents and freshly distilled before use. Reactions were monitored by Thin-layer chromatography (TLC) (Silica gel 60 F254, Merck KGaA, Darmstadt, Germany) and visualization was carried out by short wavelength UV light (254 nm). Flash column chromatography was performed using Silica Gel 60 M (40−63 µm, Machery Nagel GmbH & Co., Düren, Germany). TGA and FE-SEM, images were taken with Shimadzu TGA-60 Thermal Analyzer and scanning electron microscope (SEM) with field emission gun (Model: JSM 7100F), equipped with EDXS of SDD (Silicon drift detector) and STEM (Scanning Transmission Electron Microscope) detector respectively. Melting points were obtained on a Fisatom 430D apparatus and uncorrected. Infrared spectra were recorded on an FT-IR Thermo Nicolet IS-50 apparatus operated in the ATR mode (32 scans) (resolution 4 $cm^{-1}$). $^{1}H$ and $^{13}C$ NMR spectra were acquired on a Bruker Advance NEO spectrometer operating at 500 MHz, employing a direct broadband probe at 125 MHz in $CDCl_3$ or DMSO-$d_6$ at 25 °C. Chemical shifts (δ) are reported in parts per million relative to the residual solvent signals, and coupling constants (*J*) are reported in hertz. Multiplicities are described as brs = broad signal, s = singlet, d = doublet, t = triplet, q = quartet, dd = doublet of doublets, dt = doublet of triplets, and m = multiplet. APPI-Q-TOFMS measurements were obtained on a mass spectrometer equipped with an automatic syringe pump for sample injection.

## 2. Chitosan-$SO_3H$ synthesis and characterization

Chitosan-$SO_3H$ (CS-$SO_3H$) was prepared according to a literature method [29]. Chlorosulfonic acid (2 mL) was added dropwise at 0 °C for 1 h to a magnetically stirred suspension of chitosan (1.00 g) in dry dichloromethane (10 mL). After complete addition, the mixture was stirred for another 2 h at room temperature until HCl was removed from the reaction vessel. The mixture was then filtered and washed several times with methanol until obtaining neutral pH, followed by drying at room temperature to obtain chitosan-$SO_3H$ as a white solid.

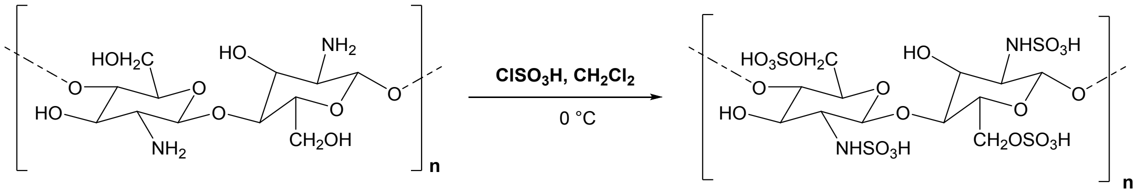


**Scheme S1.** CS-$SO_3H$ preparation.

**Catalyst characterization.**

**FT-IR catalyst analysis:**

The absorption band at 1642 $cm^{-1}$ is due to N-H bending vibration in the chitosan FT-IR spectra. The peaks at 1064 $cm^{-1}$ and 1024 $cm^{-1}$ are due to the stretching vibrations of C-O bonds. Concerning modified chitosan (CS-$SO_3H$) FT-IR spectra, the characteristic bands at 1205 $cm^{-1}$and 1084 $cm^{-1}$ are due to-S=O stretching bands of -$SO_3H$ in –O-$SO_3H$ and NH-$SO_3H$ groups, while the peak at 790 $cm^{-1}$ is due to the stretching vibration of the S-N bond in -HN-$SO_3H$.

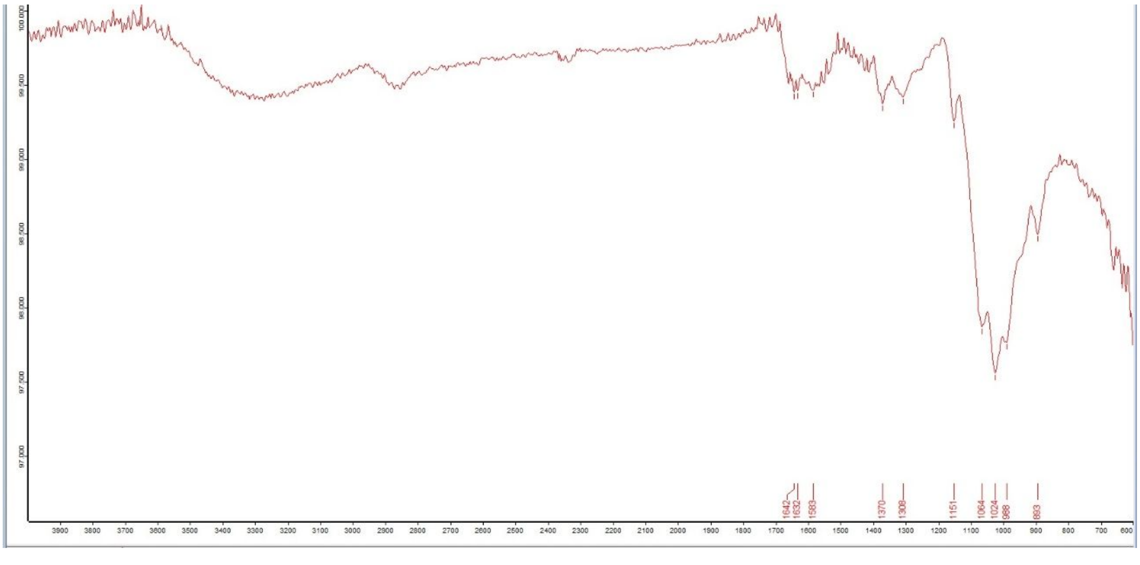


**Figure S1: Chitosan FT-IR spectra.**

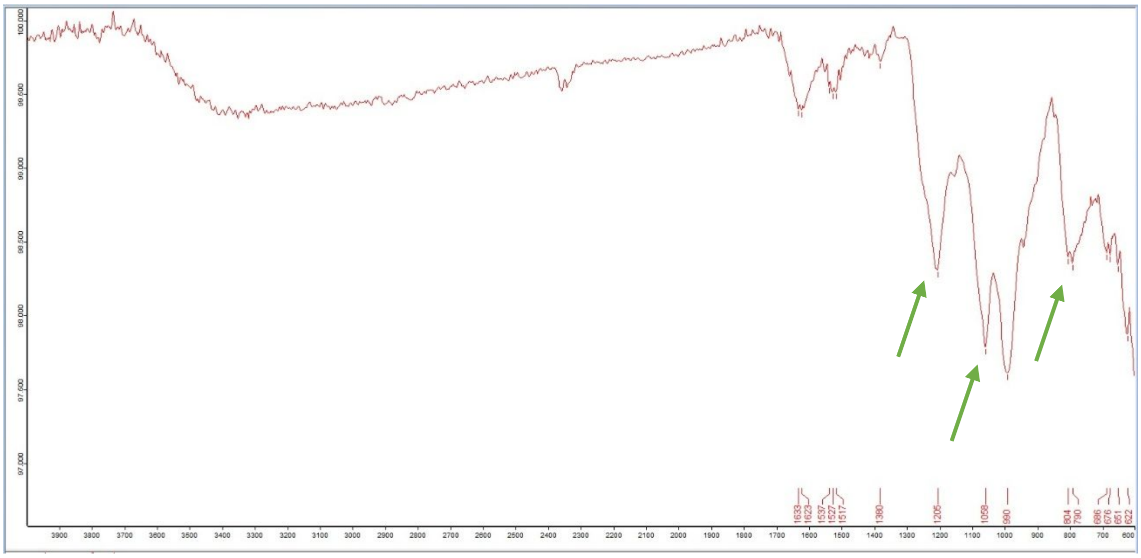


**Figure S2: Chitosan-$SO_3H$ FT-IR spectra.**

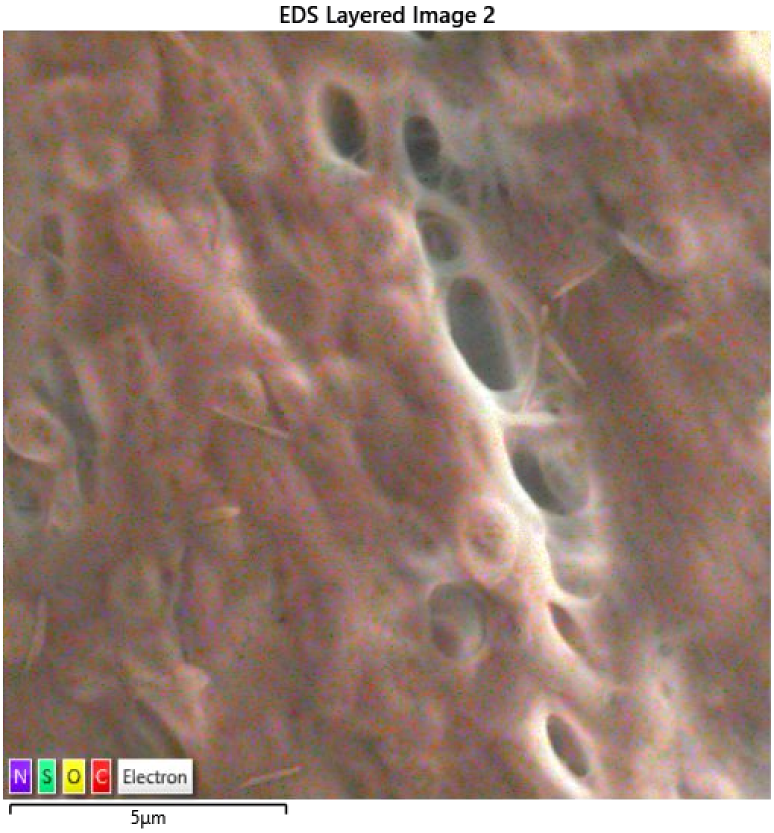

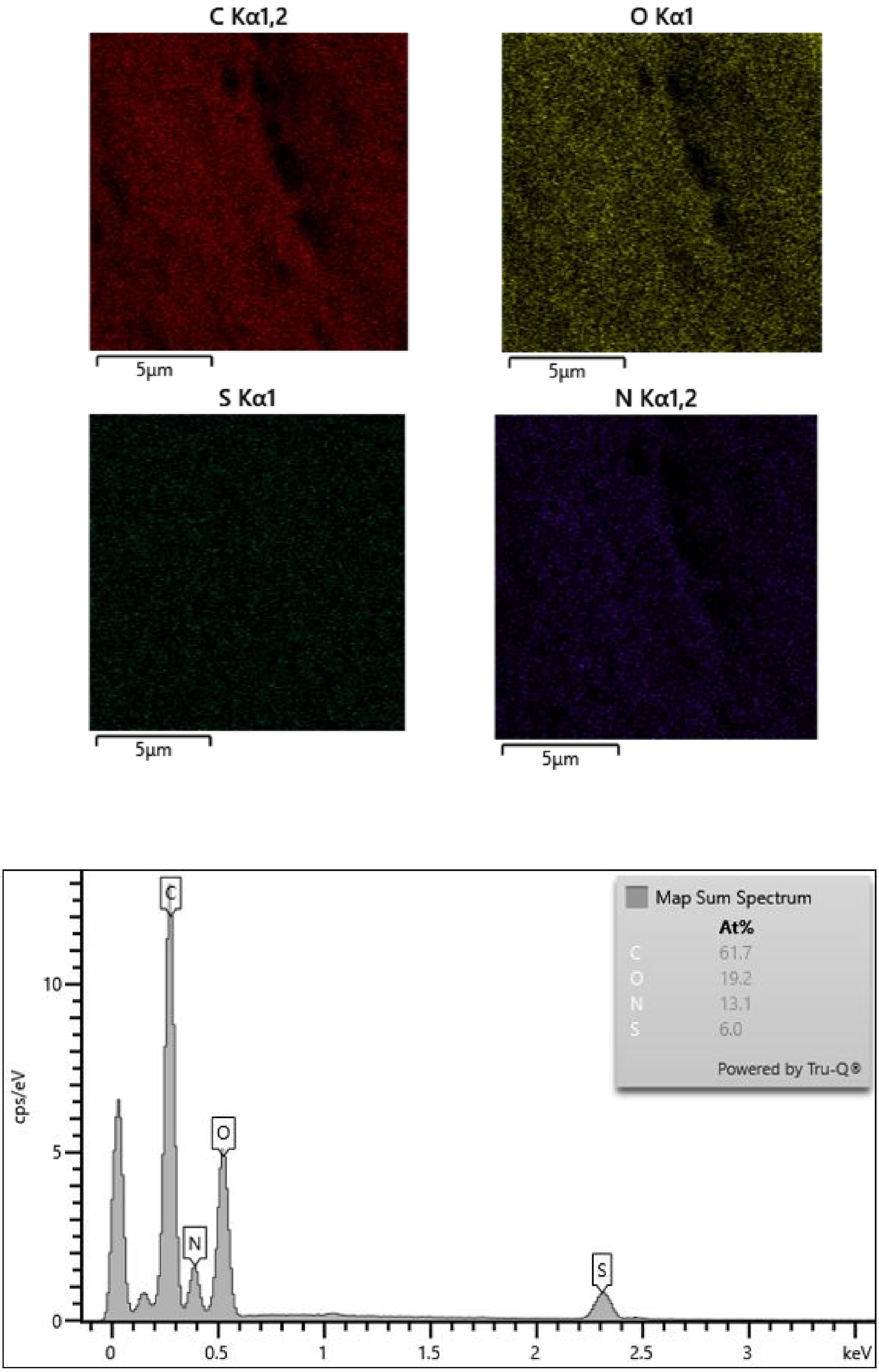


**Figure S3: Chitosan-$SO_3H$ EDS spectra.**

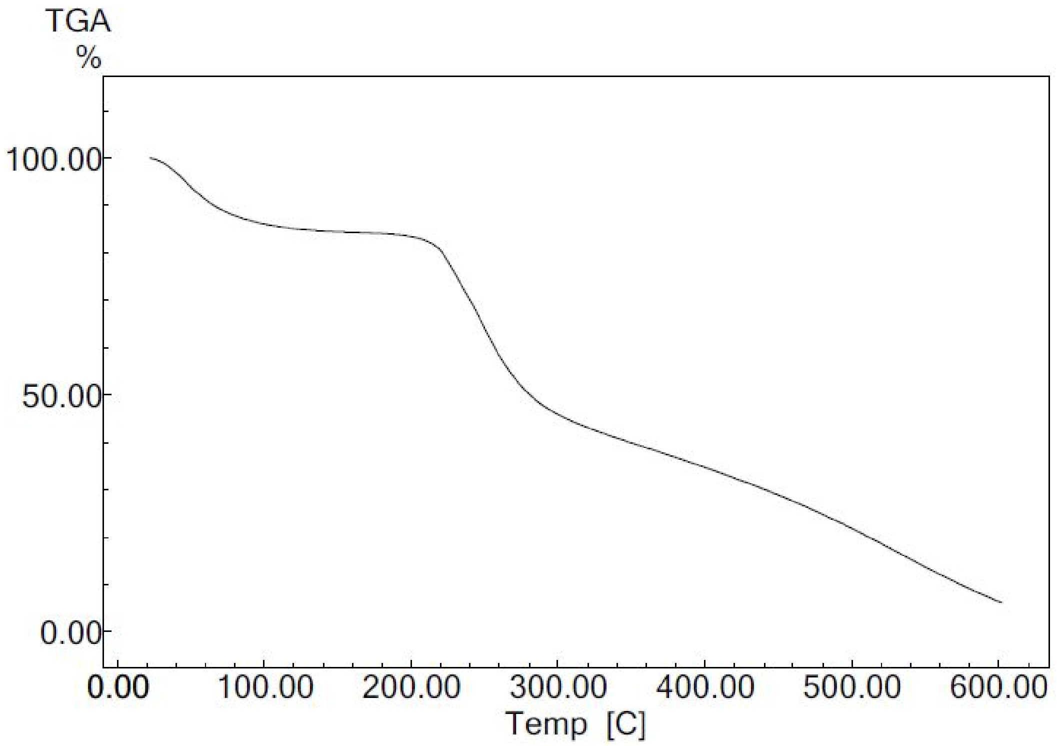


**Figure S4: TGA curve of CS- $SO_3H$.**

## 3. Scale-up Experiment

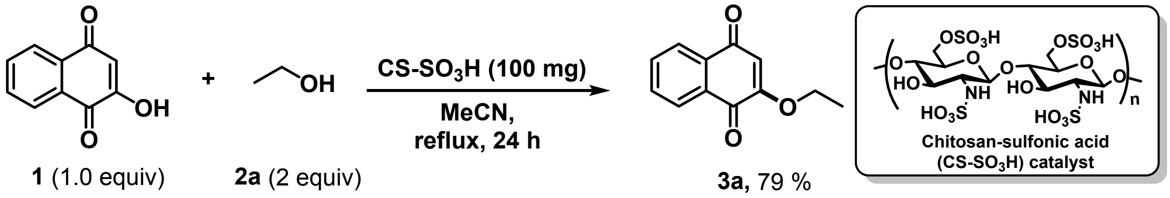


**Scheme S2.** Scale-up synthesis of **3a**.

Lawsone (5.0 mmol, 1.0 equiv.), ethyl alcohol (1.0 mmol, 2.0 equiv.), and 0.500 g of chitosan-$SO_3H$ were mixed in a 25 mL round bottom flask containing a magnetic stir bar. The mixture was then dissolved in 50.0 mL of acetonitrile and refluxed at 82 °C. Reagent consumption was monitored by thin-layer chromatography (TLC). After 24 h of reaction, product formation was observed, and the reaction was terminated. The reaction mixture was then cooled to room temperature and the product purified by flash column chromatography (10-30% EtOAc in hexane), providing compound **3a** as a light brown solid (797 mg, 79%).

## 4. General Procedure for lawsone *O*-alkylation.

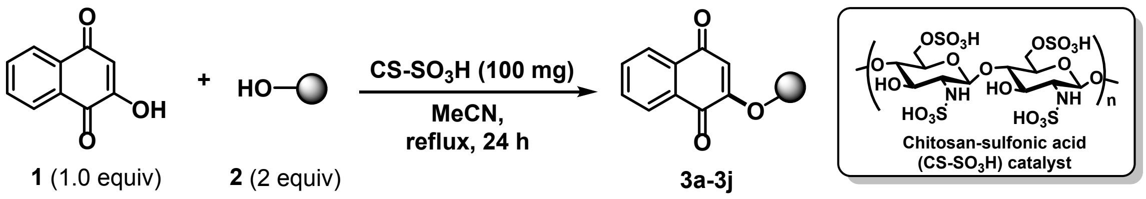


**Scheme S3.** Synthesis of 2-alkoxy-1,4-naphthoquinone.

Lawsone (1.0 mmol, 1.0 equiv.), the appropriate alcohol (2.0 mmol, 2.0 equiv.), and 0.100 g of chitosan-$SO_3H$ were mixed in a 25 mL round bottom flask containing a magnetic stir bar. The mixture was then dissolved in 10.0 mL of acetonitrile and refluxed at 82 °C. Reagent consumption was monitored by TLC. After 24 h of reaction, product formation was observed, and the reaction was terminated. The reaction mixture was then cooled to room temperature and the product purified by flash column chromatography (within the eluent indicated in each case), providing the respective product 2-alkoxy-1,4-naphthoquinone **3a-j**

## 5. Compound Characterization Data

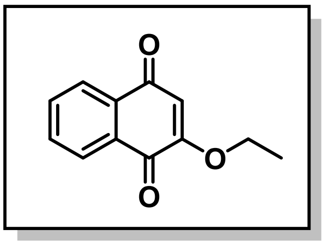

***2-ethoxynaphthalene-1,4-dione (3a):***

Product **3a** was prepared according to the general process and was purified by flash chromatography on silica gel (EtOAc/hexane 70/30%), resulting in a light brown solid (173 mg, 86% yield). **mp**: 115-118 °C. **$^1$H NMR** (500 MHz, $CDCl_3$) δ 8.13 – 8.01 (m, 2H), 7.75 – 7.64 (m, 2H), 6.13 (s, 1H), 4.08 (q, *J* = 7.0 Hz, 2H), 1.51 (t, *J* = 7.0 Hz, 3H). **$^{13}$C NMR** (126 MHz, $CDCl_3$) δ 185.02, 180.18, 159.69, 134.23, 133.27, 131.99, 131.13, 126.68, 126.10, 110.20, 65.34, 13.93. **IR** (ATR, vmax/cm$^{-1}$): 3073, 2987, 1678, 1654, 1608, 1242, 1206, 1044, 817. **HRMS (ESI):** m/z calc. for $C_{12}H_{10}NaO_3$ $[M+Na]^+$ 225.0528, found 225.0514.

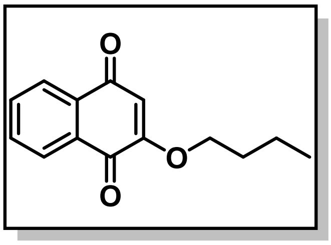

***2-butoxynaphthalene-1,4-dione (3b):***

Product **3b** was prepared according to the general process and was purified by flash chromatography on silica gel (EtOAc/hexane 70/30%), resulting in an orange solid (223 mg, 97% yield). **mp**: 108 – 109 °C. **$^1$H NMR** (500 MHz, $CDCl_3$) δ 8.13 – 8.02 (m, 2H), 7.75 – 7.65 (m, 2H), 6.14 (s, 1H), 4.00 (t, $J$ = 6.6 Hz, 2H), 1.94 – 1.80 (m, 2H), 1.58 – 1.43 (m, 2H), 0.98 (t, $J$ = 7.4 Hz, 3H). **$^{13}$C NMR** (126 MHz, $CDCl_3$) δ 185.06, 180.15, 159.91, 134.20, 133.24, 132.02, 131.18, 126.66, 126.09, 110.18, 69.38, 30.24, 19.12, 13.70. **FT-IR** (ATR, vmax/cm$^{-1}$): 3054, 2955, 1681, 1605, 1362, 1245, 1042, 738. **HRMS (ESI):** m/z calc. for $C_{14}H_{14}NaO_3$ [M+Na]$^+$ 253.0841, found 253.0830.

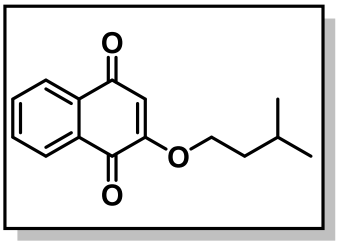

***2-(isopentyloxy)naphthalene-1,4-dione (3c):***

Product **3c** was prepared according to the general process and was purified by flash chromatography on silica gel (EtOAc/hexane 70/30%), resulting in a yellow solid (217 mg, 89% yield). **mp**: 71 – 72 °C. **$^1$H NMR** (500 MHz, $CDCl_3$) δ 8.13 – 8.03 (m, 2H), 7.75 – 7.64 (m, 2H), 6.13 (s, 1H), 3.83 (d, $J$ = 6.1 Hz, 1H), 3.77 (d, $J$ = 6.7 Hz, 1H), 2.08 – 1.94 (m, 1H), 1.65 – 1.49 (m, 1H), 1.30 (dt, $J$ = 13.6, 7.6 Hz, 1H), 1.07 – 0.92 (m, 6H). **$^{13}$C NMR** (126 MHz, $CDCl_3$) δ 185.08, 180.08, 160.03, 134.20, 134.17, 133.25, 133.23, 132.04, 131.22, 110.15, 74.24, 33.98, 26.01, 16.37, 11.19. **FT-IR** (ATR, vmax/cm$^{-1}$): 3057, 2958, 1680, 1650, 1247, 1038, 725. **HRMS (ESI):** m/z calc. for $C_{15}H_{16}NaO_3$ [M+Na]$^+$ 267.0997, found 267.0983.

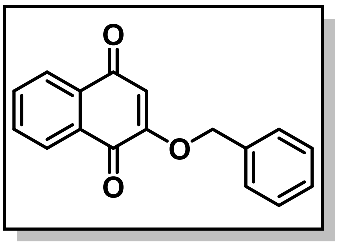

***2-(benzyloxy)naphthalene-1,4-dione (3d):***

Product **3d** was prepared according to the general process and was purified by flash chromatography on silica gel (EtOAc/hexane 07/93%), resulting in a yellow solid (216 mg, 82% yield). **mp**: 148 - 149 °C. **$^1$H NMR** (500 MHz, $CDCl_3$) δ 8.17 – 8.01 (m, 2H), 7.75 – 7.65 (m, 2H), 7.48 – 7.31 (m, 5H), 6.22 (s, 1H), 5.12 (s, 2H). **$^{13}$C NMR** (126 MHz, $CDCl_3$) δ 184.91, 180.03, 159.31, 134.27, 134.18, 133.35, 131.96, 131.16, 128.90, 128.77, 127.63, 126.69,

126.15, 111.20, 71.12. **FT-IR** (ATR, vmax/cm$^{-1}$): 3053, 1681, 1651, 1247, 1011, 747. **HRMS (ESI):** m/z calc. for $C_{17}H_{12}NaO_3$ $[M+Na]^+$ 287.0684, found 287.0687.

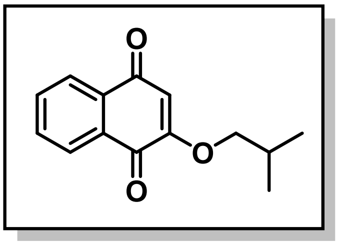

***2-isobutoxynaphthalene-1,4-dione (3e):***

Product **3e** was prepared according to the general process and was purified by flash chromatography on silica gel (EtOAc/hexane 10/90%), resulting in a light brown solid (177 mg, 77% yield). **mp**: 84 -88 °C. **$^1$H NMR** (500 MHz, $CDCl_3$) δ 8.13 – 8.03 (m, 2H), 7.76 – 7.65 (m, 2H), 6.13 (s, 1H), 3.75 (d, *J* = 6.7 Hz, 2H), 2.26-2.18 (m, 1H), 1.05 (d, *J* = 6.8 Hz, 6H). **$^{13}$C NMR** (126 MHz, $CDCl_3$) δ 185.07, 180.08, 159.96, 134.17, 133.24, 132.03, 131.21, 126.63, 126.09, 110.18, 75.59, 27.64, 19.11. **FT-IR** (ATR, vmax/cm$^{-1}$): 3055, 2960, 1652, 1596, 1266, 1038, 721. **HRMS (ESI):** m/z calc. for $C_{14}H_{14}NaO_3$ $[M+Na]^+$ 253.0841, found 253.0848.

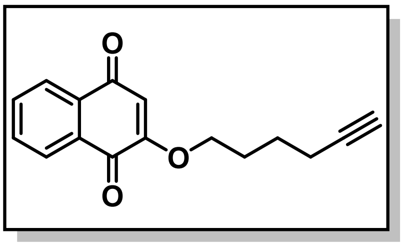

***2-(hex-5-yn-1-yloxy)naphthalene-1,4-dione (3f):***

Product **3f** was prepared according to the general process and was purified by flash chromatography on silica gel (EtOAc/hexane 10/90%), resulting in a yellow solid (124 mg, 49% yield). **mp**: 89 – 91 °C. **$^1$H NMR** (500 MHz, $CDCl_3$) δ 8.16 – 7.99 (m, 2H), 7.79 – 7.62 (m, 2H), 6.14 (s, 1H), 4.03 (t, *J* = 6.4 Hz, 2H), 2.30-2.26 (m, 2H), 2.07 – 1.95 (m, 3H), 1.75-1.70 (m, 2H). **$^{13}$C NMR** (126 MHz, $CDCl_3$) δ 184.99, 180.04, 159.75, 134.24, 133.29, 131.99, 131.15, 126.66, 126.11, 110.25, 83.62, 69.07, 68.97, 27.29, 24.76, 18.06. **FT-IR** (ATR, vmax/cm$^{-1}$): 3286, 3244, 2956, 1631, 1605, 1248, 1026, 698. **HRMS (ESI):** m/z calc. for $C_{16}H_{14}NaO_3$ $[M+Na]^+$ 277.0841, found 277.0839.

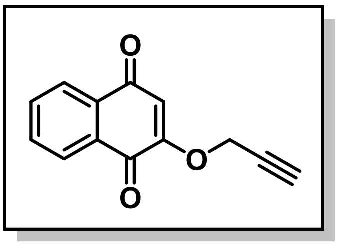

***2-(prop-2-yn-1-yloxy)naphthalene-1,4-dione (3g):***

Product **3g** was prepared according to the general process and was purified by flash chromatography on silica gel (DCM as eluent), resulting in a light yellow solid (110 mg, 52% yield). **mp**: 146 – 149 °C. **$^1$H NMR** (500 MHz, $CDCl_3$) δ 8.19 – 8.01 (m, 2H), 7.77 – 7.68 (m, 2H), 6.34 (s, 1H), 4.79 (d, *J* = 2.4 Hz, 2H), 2.65 (s, 1H). **$^{13}$C NMR** (126 MHz, $CDCl_3$) δ 183.90, 178.77, 158.00, 141.59, 138.17, 133.32,

132.41, 128.28, 127.40, 110.34, 96.43, 73.72, 68.29. **FT-IR** (ATR, vmax/cm$^{-1}$): 3250, 3053, 1680, 1603, 1261, 1244, 1040, 737.

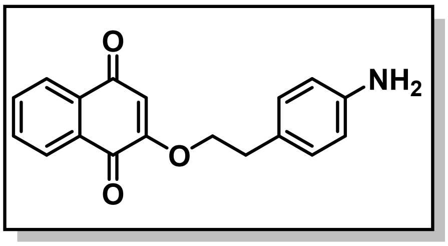

***2-(4-aminophenethoxy)naphthalene-1,4-dione (3h):***

Product **3h** was prepared according to the general process and was purified by flash chromatography on silica gel (EtOAc/hexane 10/90%), resulting in a red solid (247 mg, 61% yield). **mp**: 107 – 110 °C. **$^1$H NMR** (500 MHz, $CDCl_3$) δ δ 8.13 – 8.07 (m, 2H), 8.05 (s, 1H), 7.75 (tt, *J* = 7.5, 1.1 Hz, 1H), 7.65 (tt, *J* = 7.5, 1.1 Hz, 1H), 7.55 (s, 1H), 7.30 – 7.19 (m, 4H), 6.37 (d, *J* = 0.9 Hz, 1H), 4.39 (t, *J* = 6.9 Hz, 2H), 2.98 (t, *J* = 6.9 Hz, 2H). **$^{13}$C NMR** (126 MHz, $CDCl_3$) δ 182.85, 181.01, 159.88, 143.75, 135.08, 133.96, 133.90, 132.20, 131.33, 129.34, 129.12, 125.50, 125.14, 121.81, 102.33, 63.07, 33.37. **FT-IR** (ATR, vmax/cm$^{-1}$): 3272, 2945, 1714, 1681, 1594, 1514, 1177, 727.

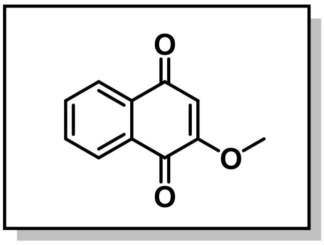

***2-methoxynaphthalene-1,4-dione (3i):***

Product **3i** was prepared according to the general process and was purified by recrystallization in ethanol, resulting in a light yellow solid (156 mg, 83% yield). **mp**: 167 – 170 °C. **$^1$H NMR** (500 MHz, $CDCl_3$) δ 8.14 – 8.01 (m, 2H), 7.78 – 7.63 (m, 2H), 6.16 (s, 1H), 3.89 (s, 3H). **$^{13}$C NMR** (126 MHz, $CDCl_3$) δ 184.85, 180.11, 160.44, 134.35, 133.36, 132.02, 131.05, 126.71, 126.19, 109.90, 56.44. **FT-IR** (ATR, vmax/cm$^{-1}$): 3048, 1679, 1602, 1241, 1043, 722.

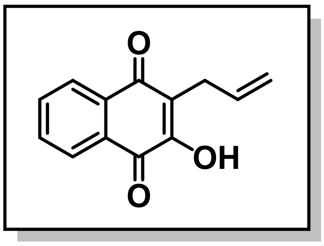

***2-allyl-3-hydroxynaphthalene-1,4-dione (3j):***

Product **3j** was prepared according to the general process and by flash chromatography on silica gel (EtOAc/hexane 30/70%), resulting in a light brown oil (117 mg, 55% yield). **$^1$H NMR** ($CDCl_3$, 500 MHz) *δ* 8.18 – 8.03 (m, 2H), 7.76 (td, *J* = 7.6, 1.4 Hz, 1H), 7.69 (td, *J* = 7.5, 1.3 Hz, 1H), 7.36 (s, 1H), 5.97 – 5.86 (m, 1H), 5.17 (dd, *J* = 17.1, 1.6 Hz, 1H), 5.05 (dd, *J* = 10.0, 1.6 Hz, 1H), 3.37 (d, *J* = 6.5 Hz, 2H).

## 6. $^{1}H$, $^{13}C$ NMR and HRMS spectra

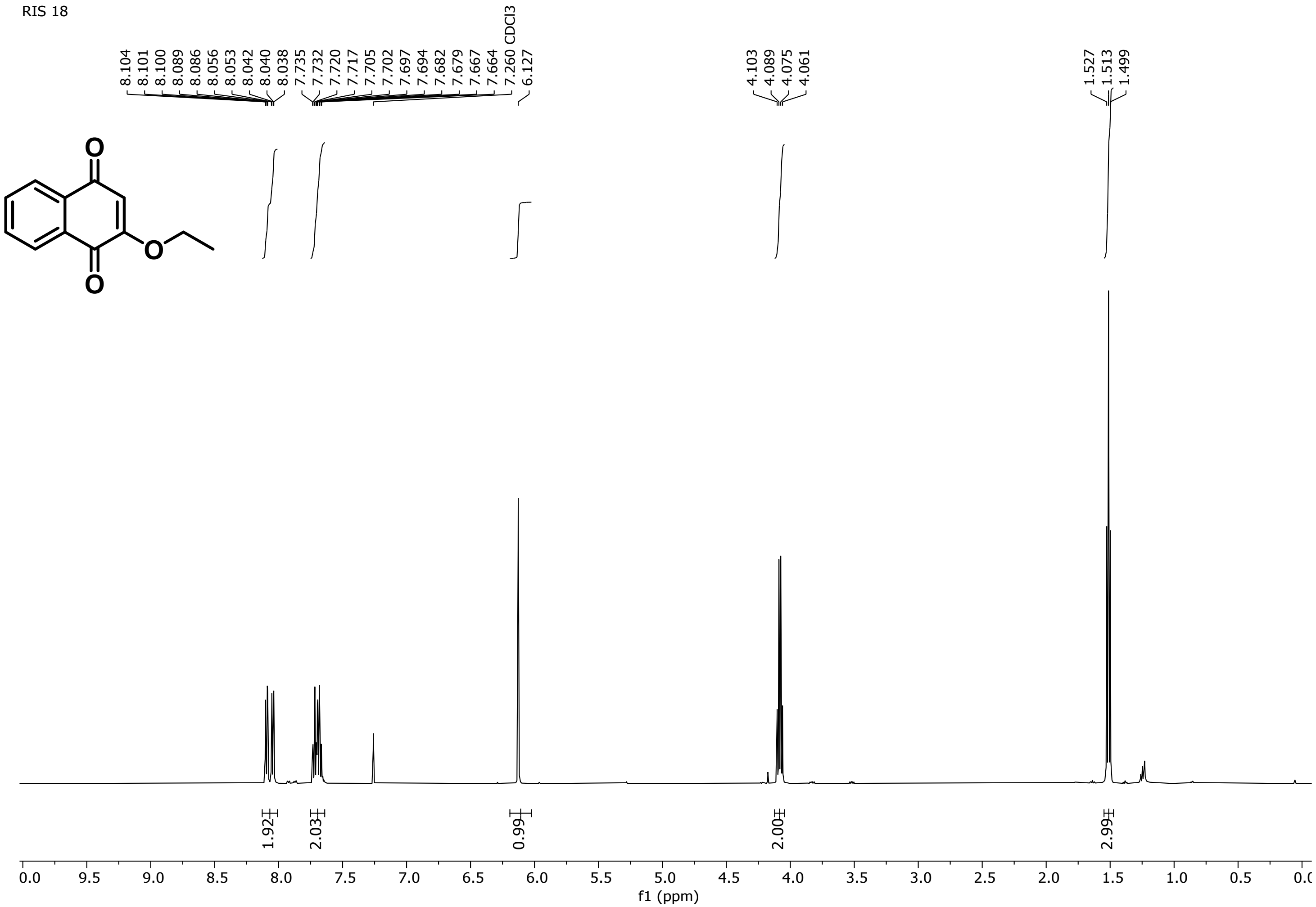


**Figure S5.** $^{1}H$ NMR spectrum of **3a** (500 MHz, $CDCl_3$).

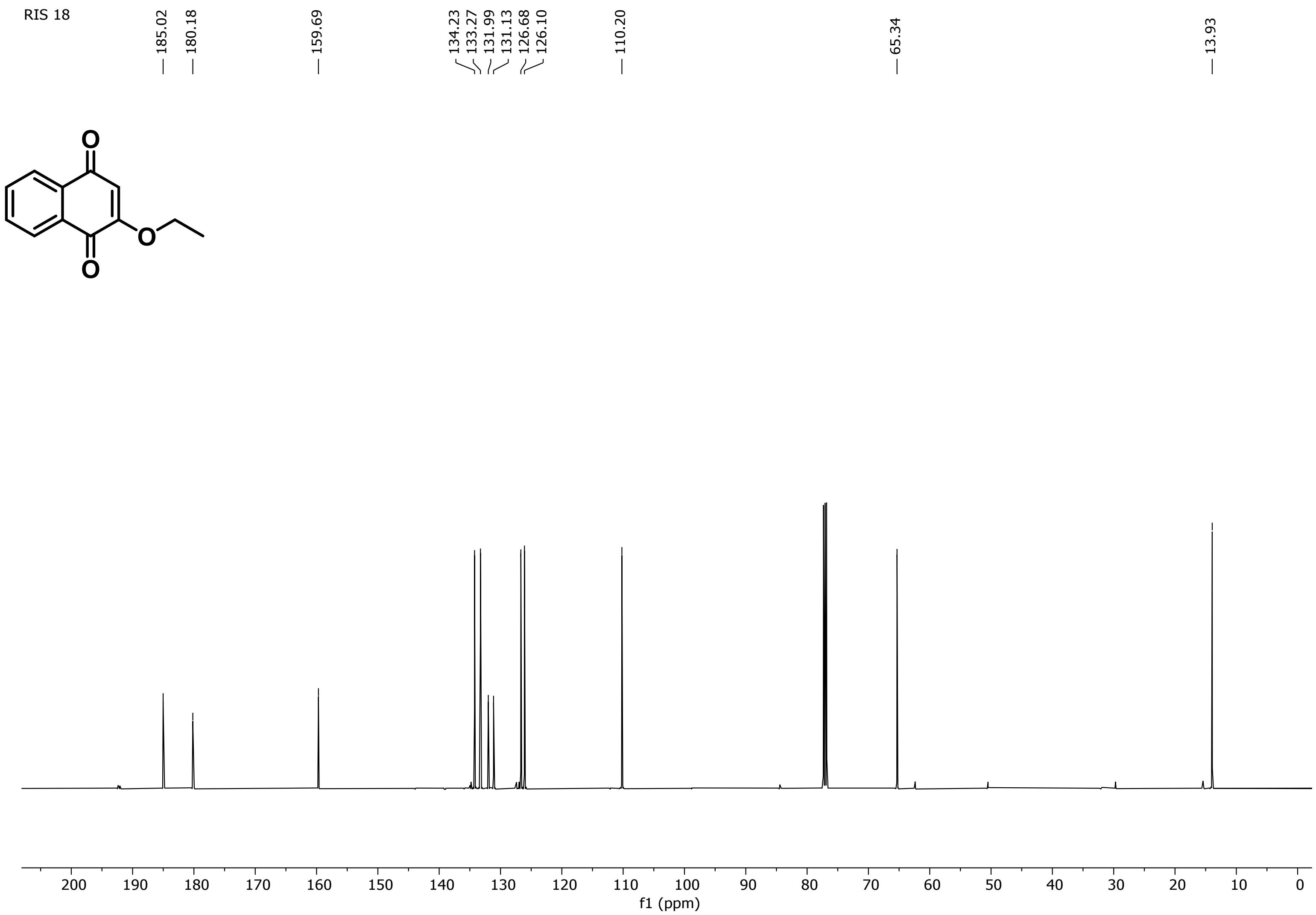


**Figure S6.** $^{13}C$ NMR spectrum of **3a** (125 MHz, $CDCl_3$).

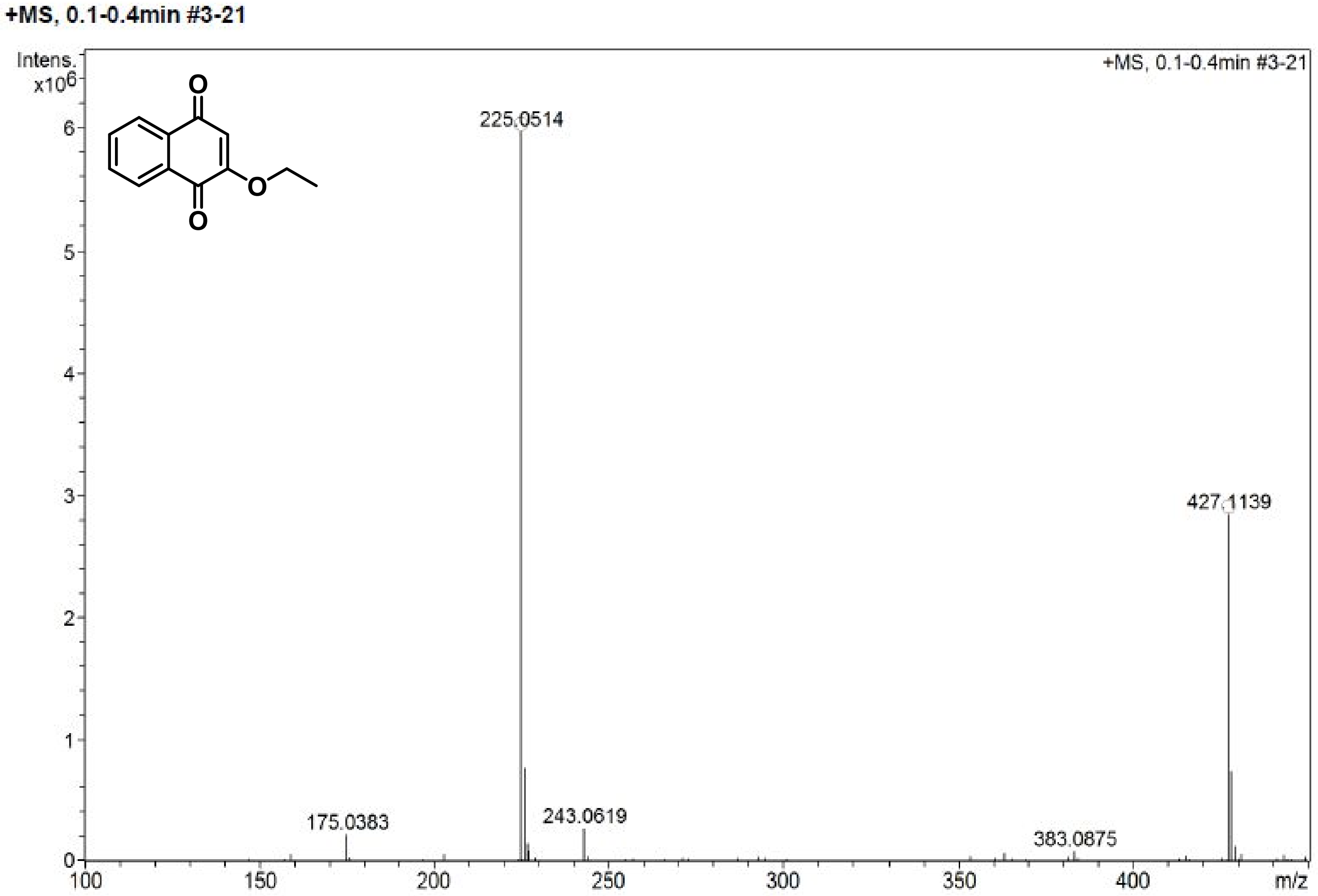


**Figure S7.** HRMS spectrum of **3a**.

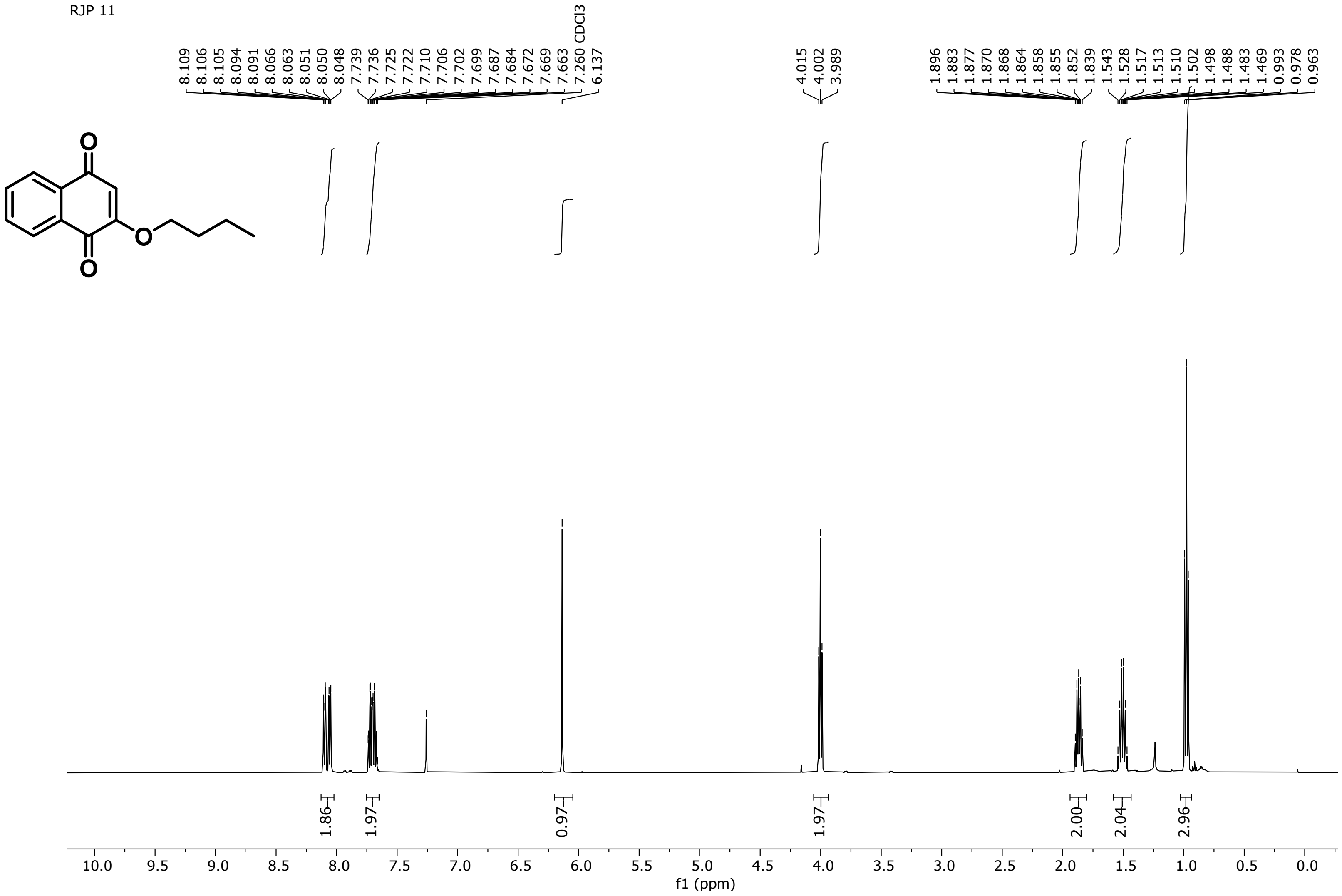


**Figure S8.** $^{1}$H NMR spectrum of **3b** (500 MHz, $CDCl_3$).

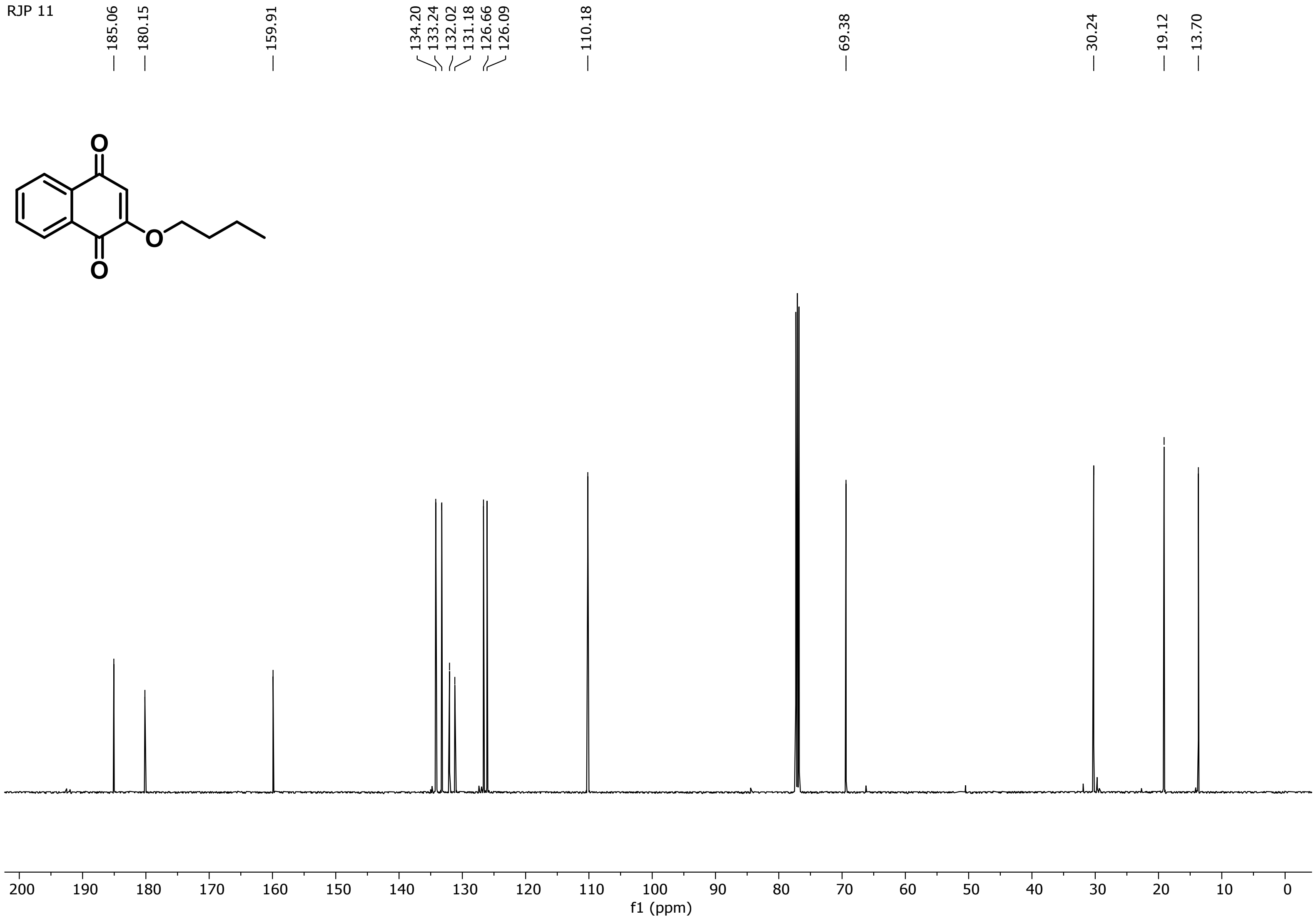


**Figure S9.** $^{13}C$ NMR spectrum of **3b** (125 MHz, $CDCl_3$).

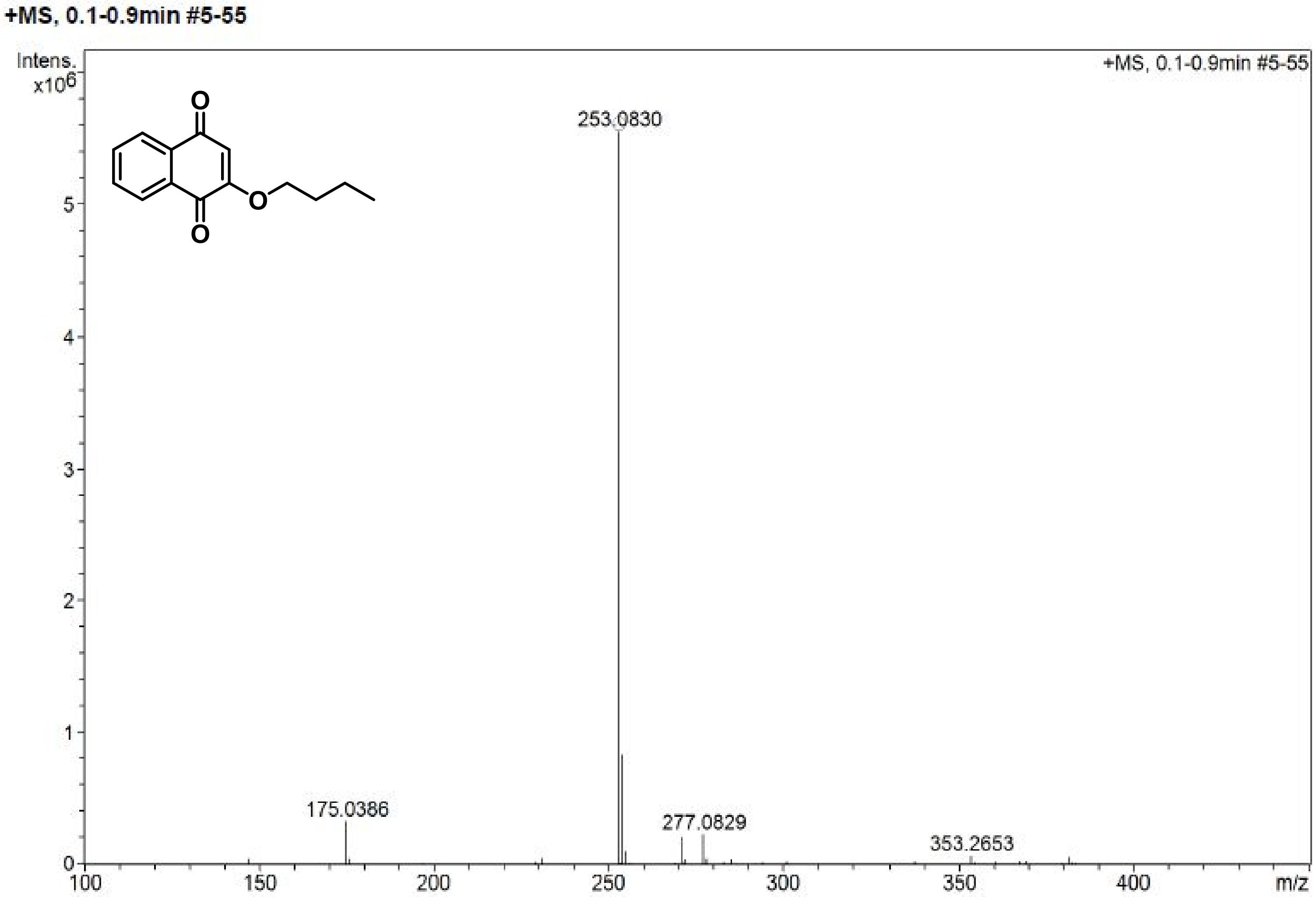


**Figure S10.** HRMS spectrum of **3b**.

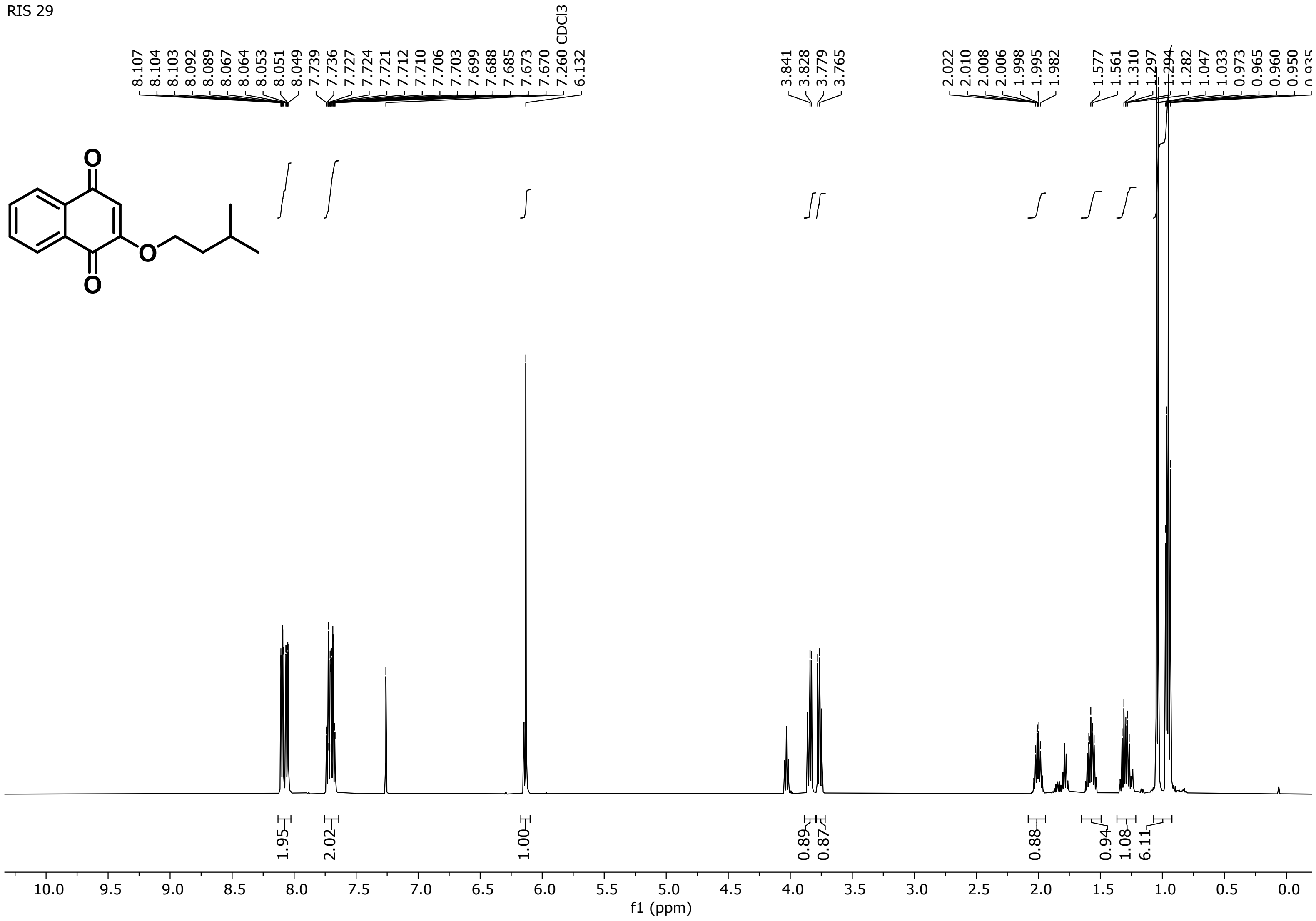


**Figure S11.** $^1$H NMR spectrum of **3c** (500 MHz, $CDCl_3$).

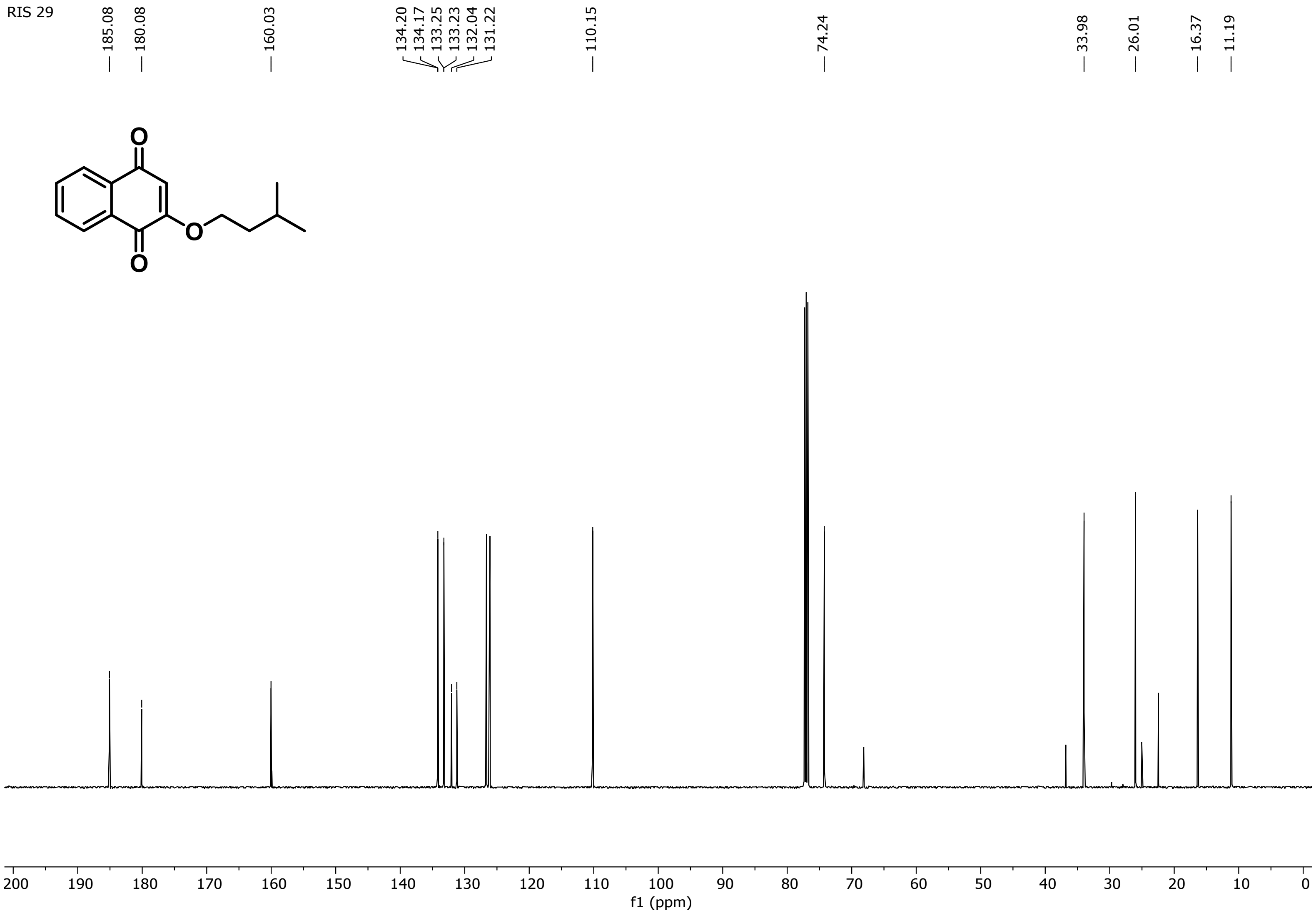


**Figure S12.** $^{13}C$ NMR spectrum of **3c** (125 MHz, $CDCl_3$).

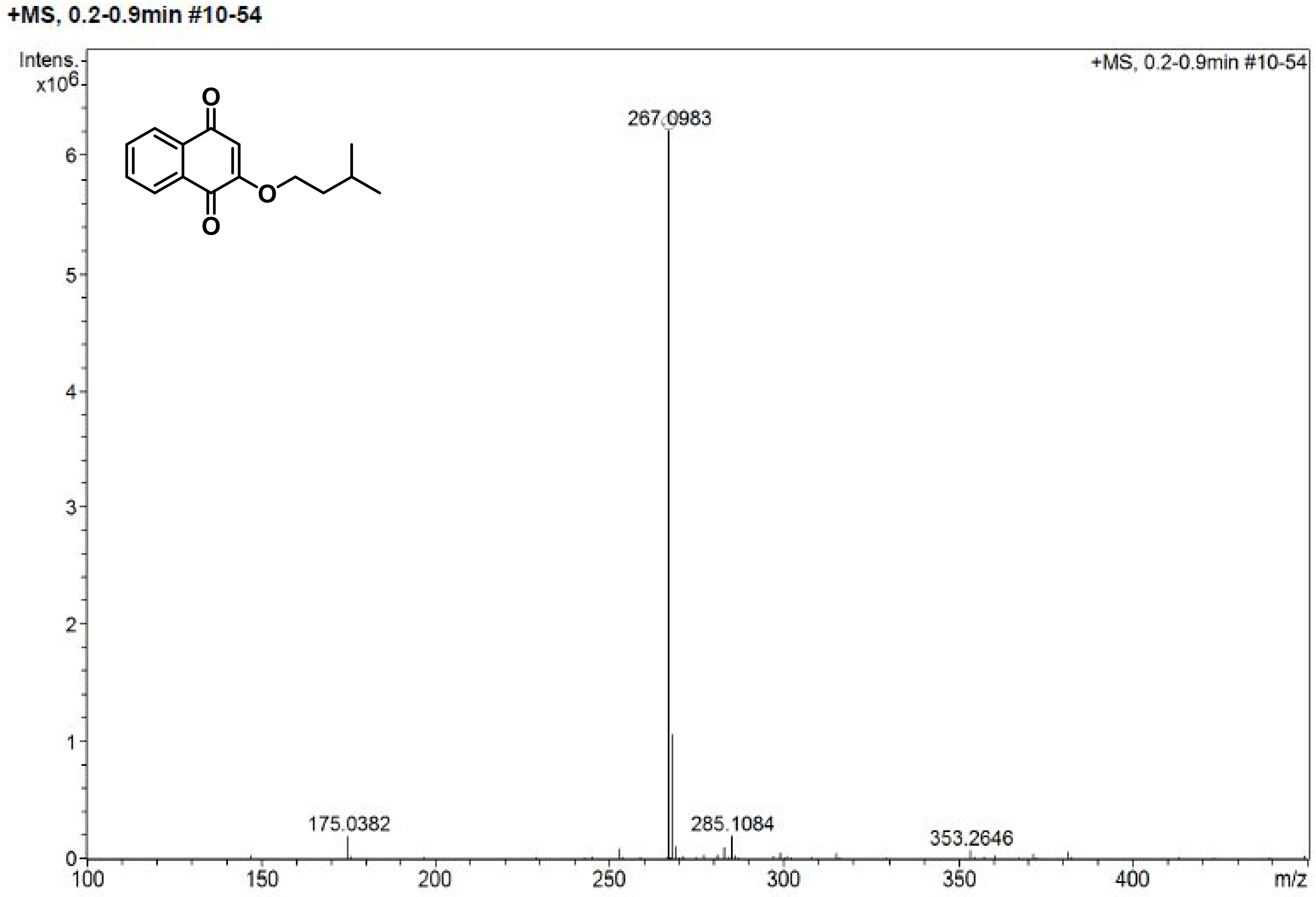


**Figure S13.** HRMS spectrum of **3c**.

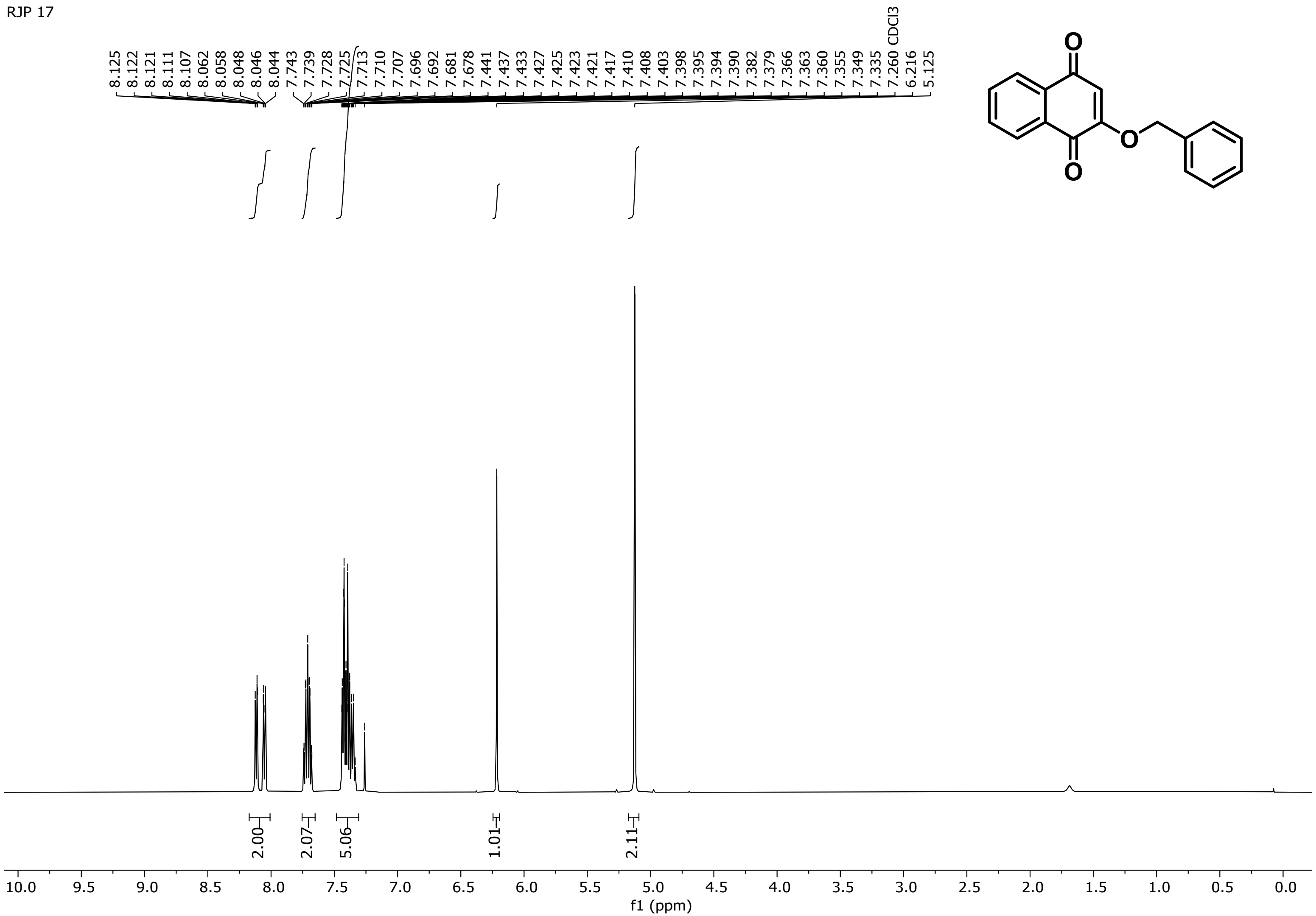


**Figure S14.** $^{1}$H NMR spectrum of **3d** (500 MHz, $CDCl_3$).

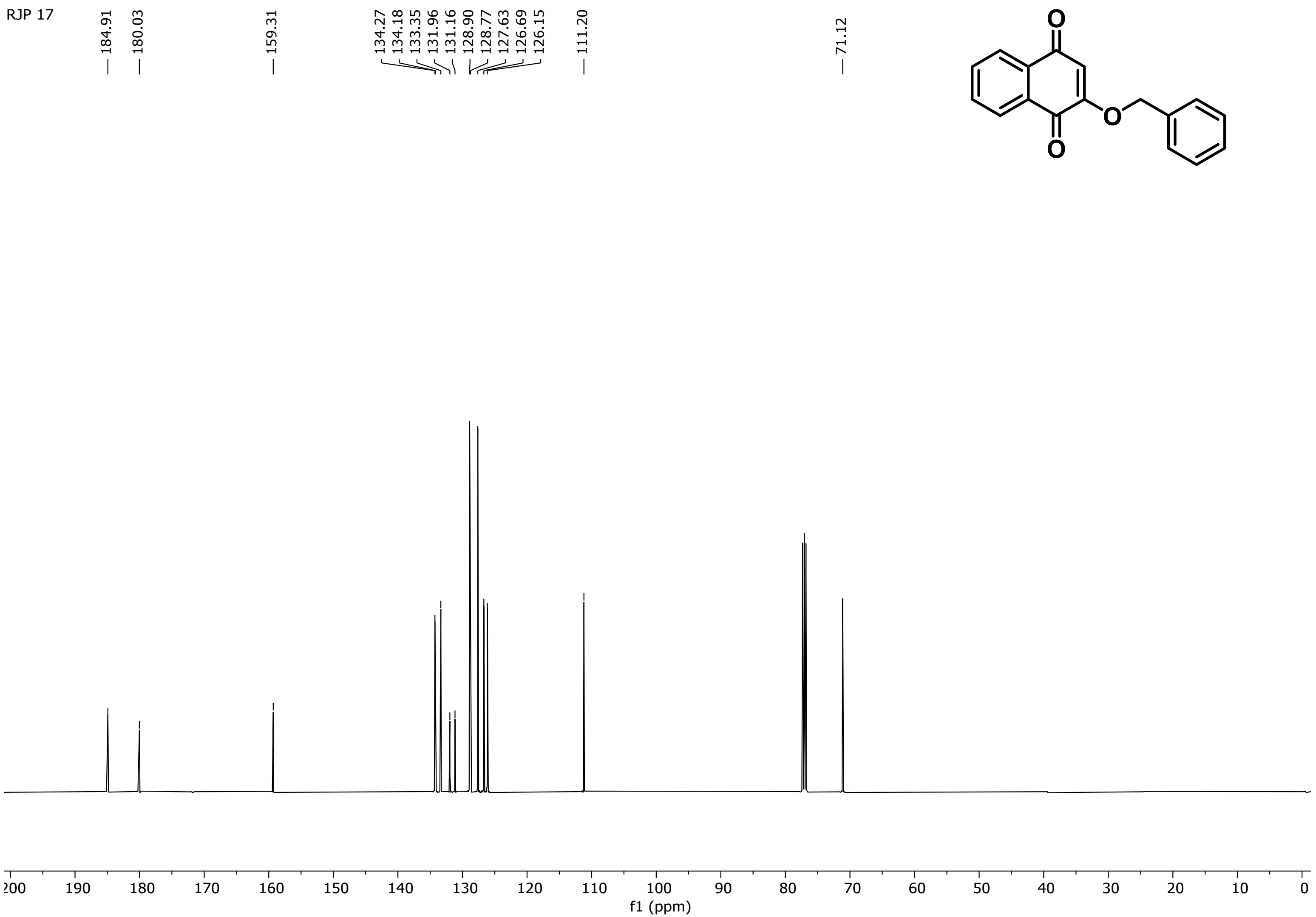


**Figure S15.** $^{13}C$ NMR spectrum of **3d** (125 MHz, $CDCl_3$).

+MS, 0.2-0.9min #11-55

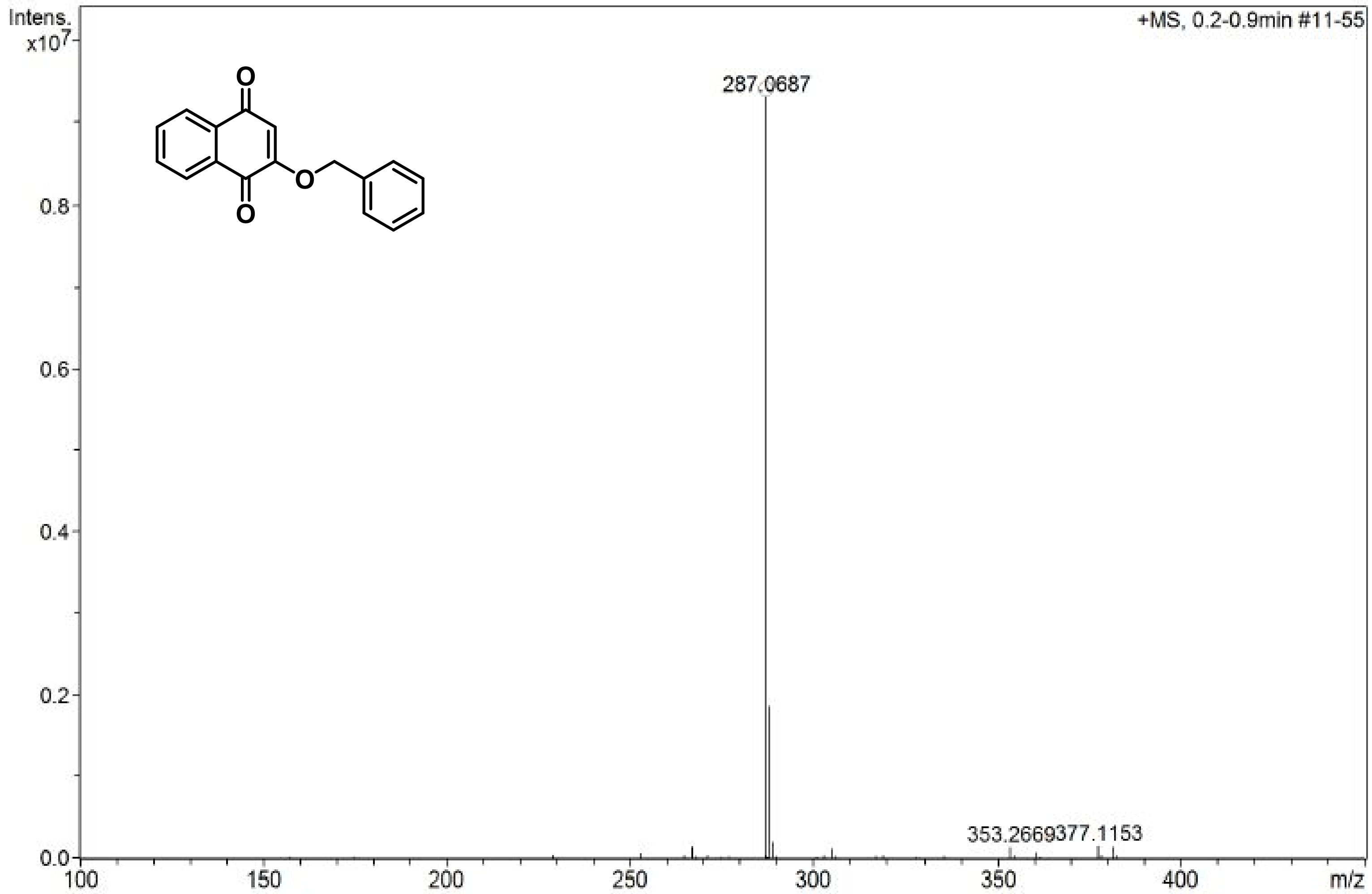


**Figure S16.** HRMS spectrum of **3d**.

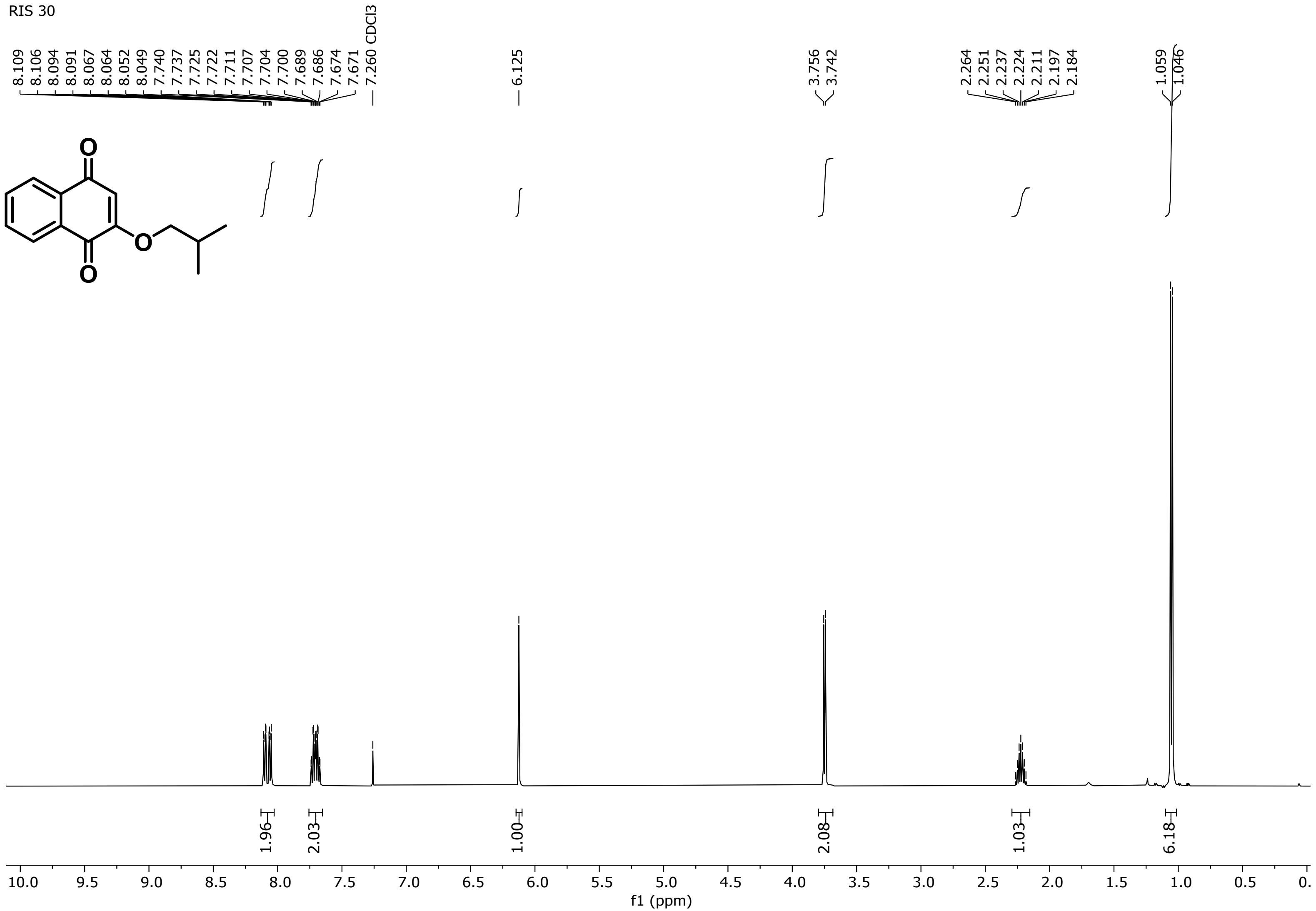


**Figure S17.** $^1$H NMR spectrum of **3e** (500 MHz, $CDCl_3$).

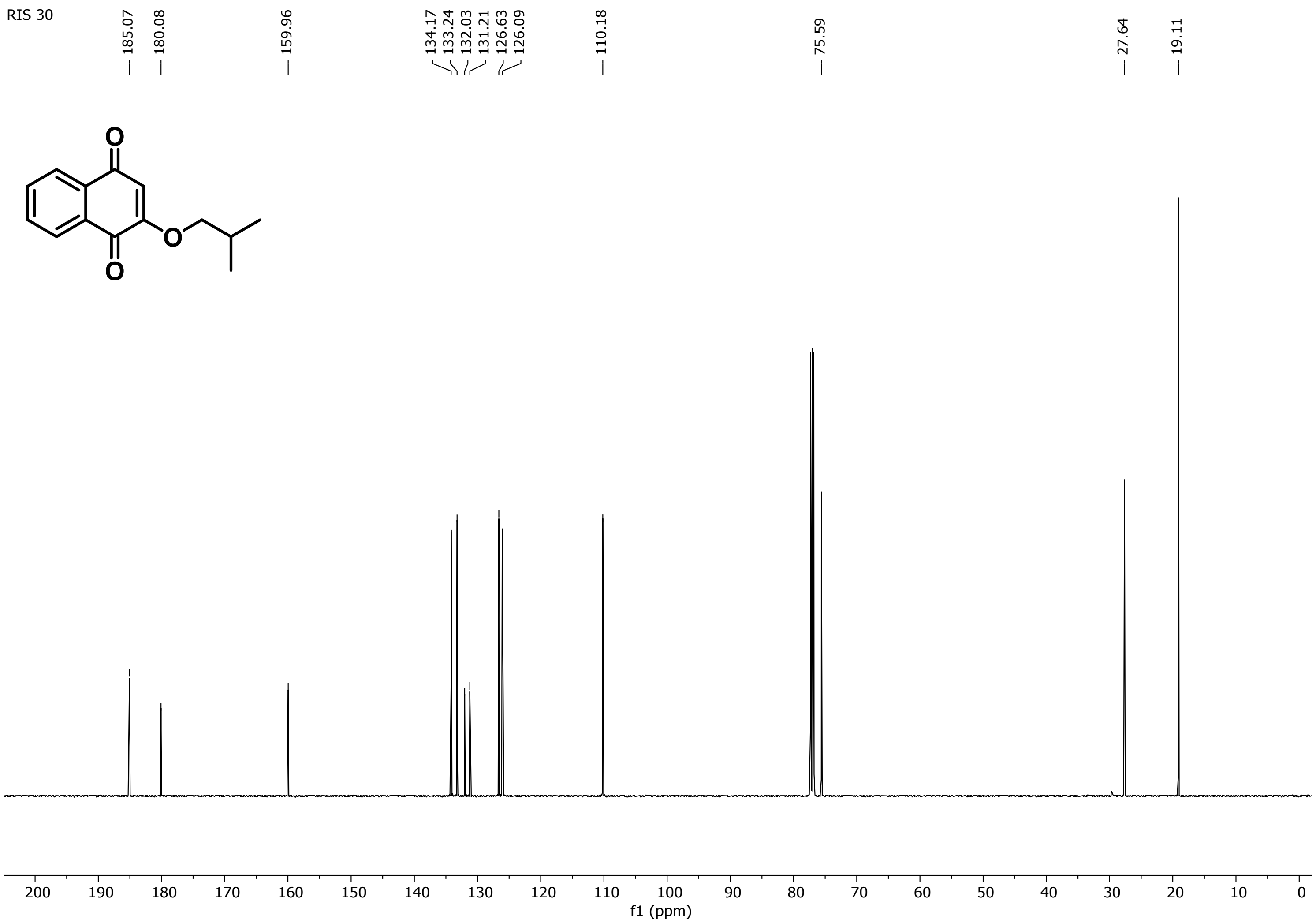


**Figure S18.** $^{13}$C NMR spectrum of **3e** (125 MHz, $CDCl_3$).

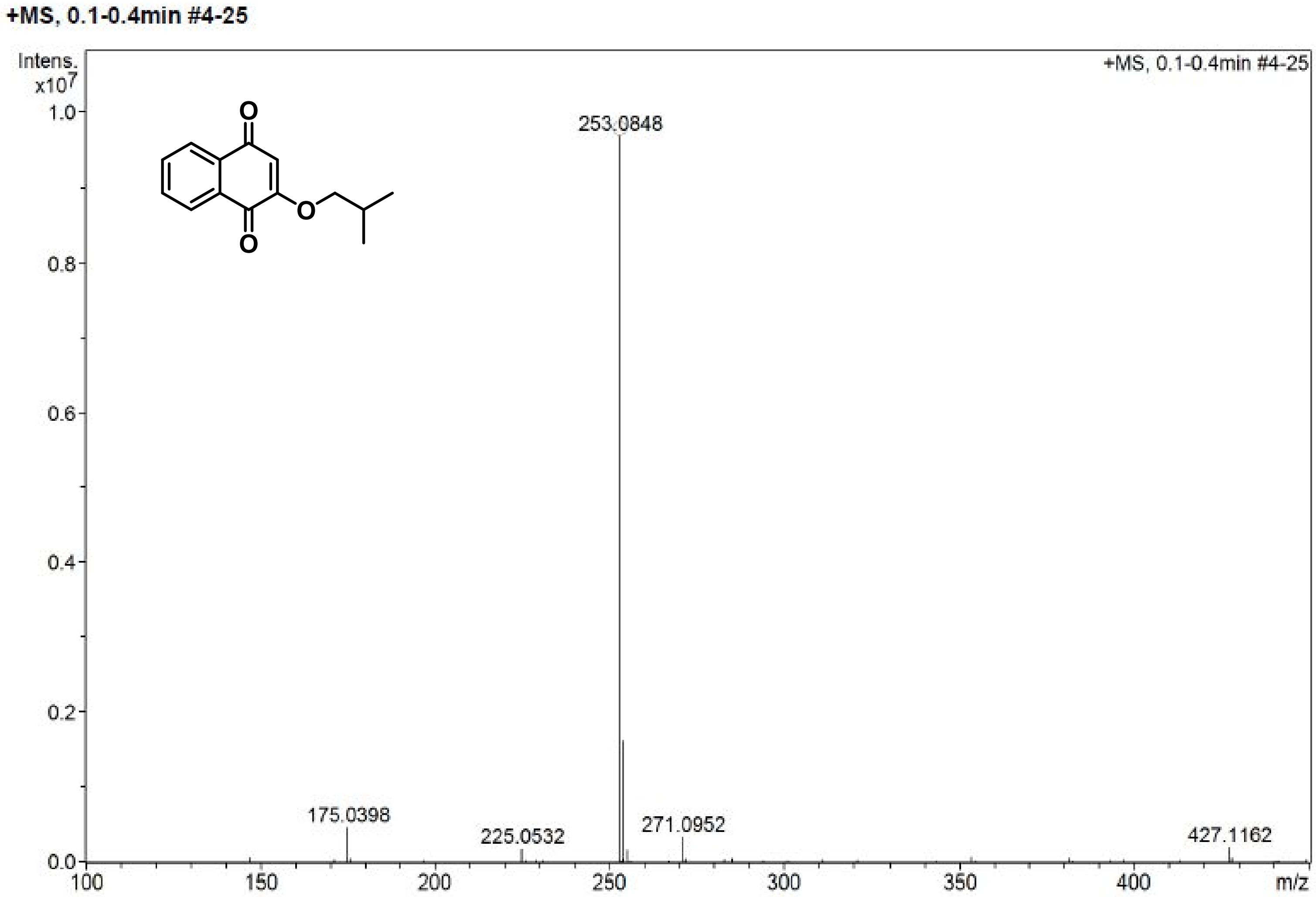


**Figure S19.** HRMS spectrum of **3e**.

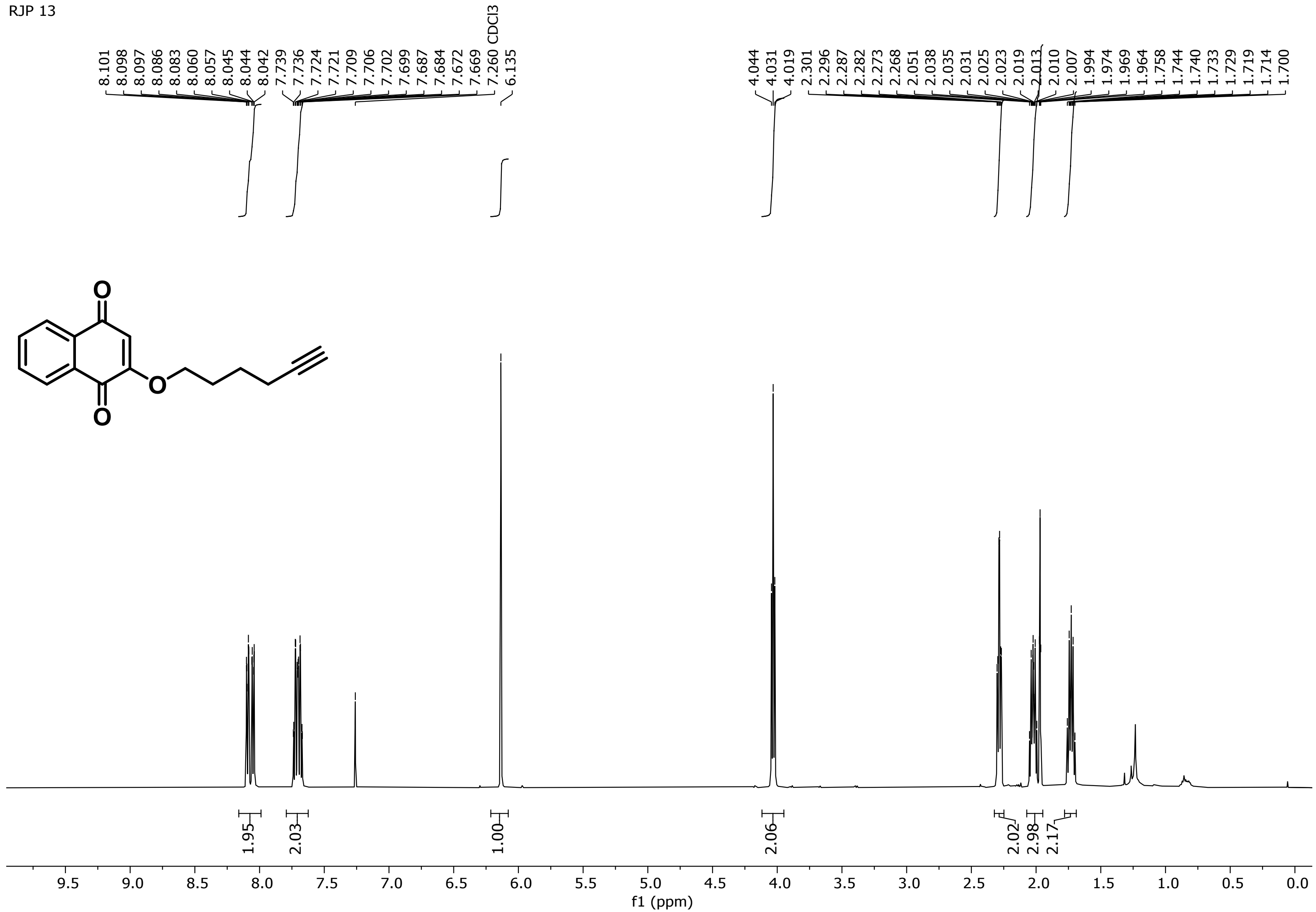


**Figure S20.** $^{1}$H NMR spectrum of **3f** (500 MHz, $CDCl_3$).

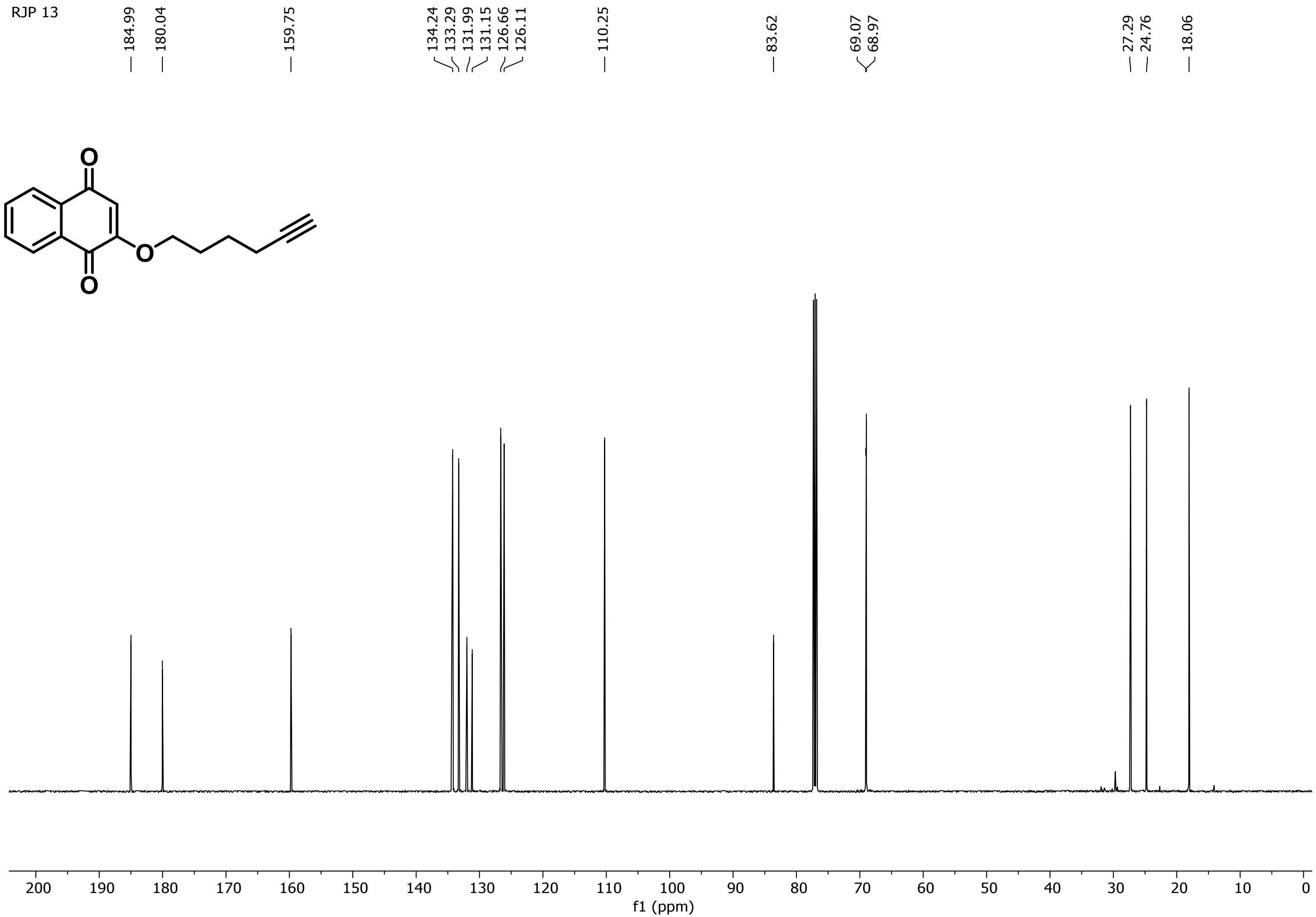


**Figure S21.** $^{13}$C NMR spectrum of **3f** (125 MHz, $CDCl_3$).

+MS, 0.1-0.9min #6-55

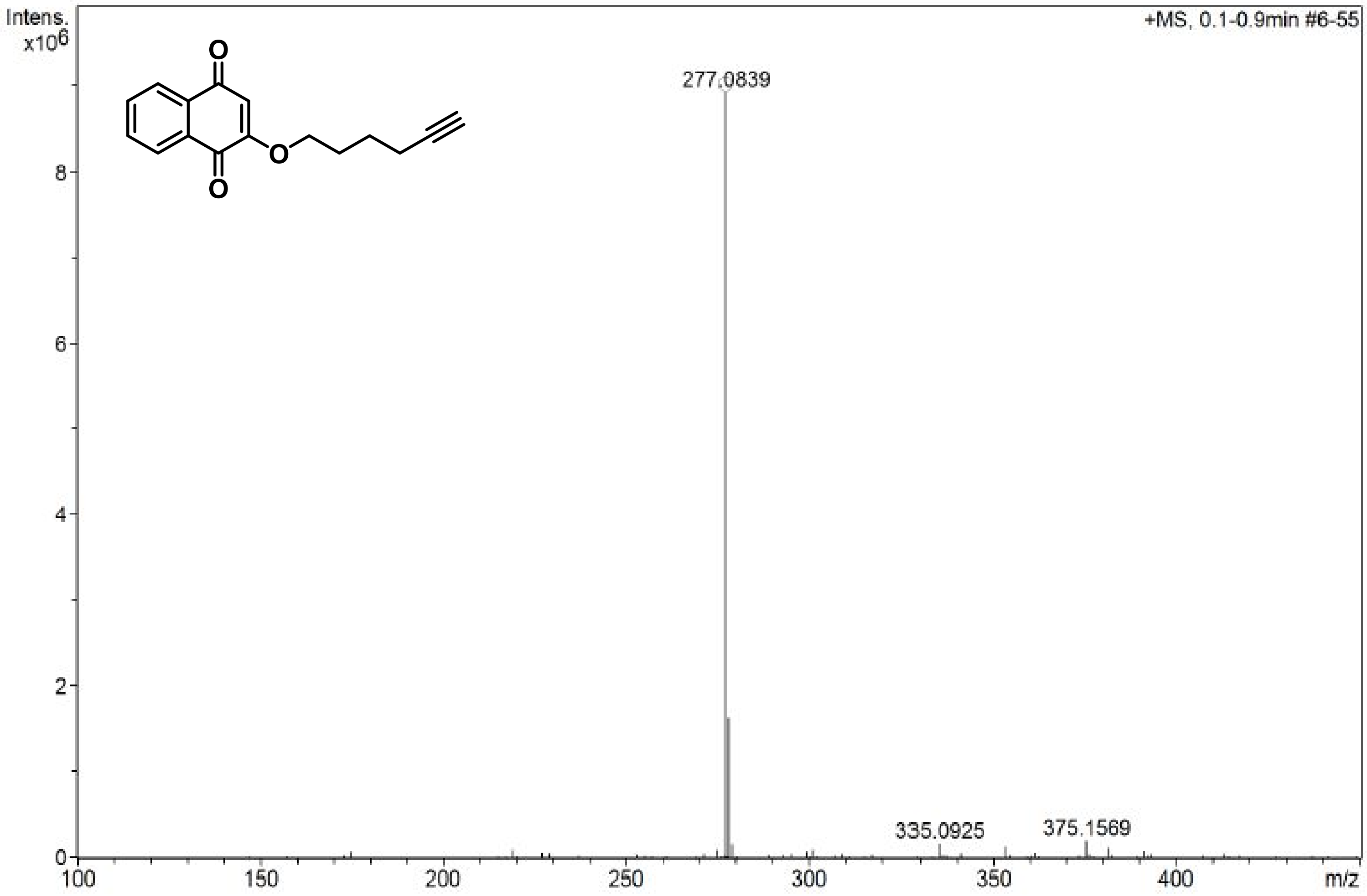


**Figure S22.** HRMS spectrum of **3f**.

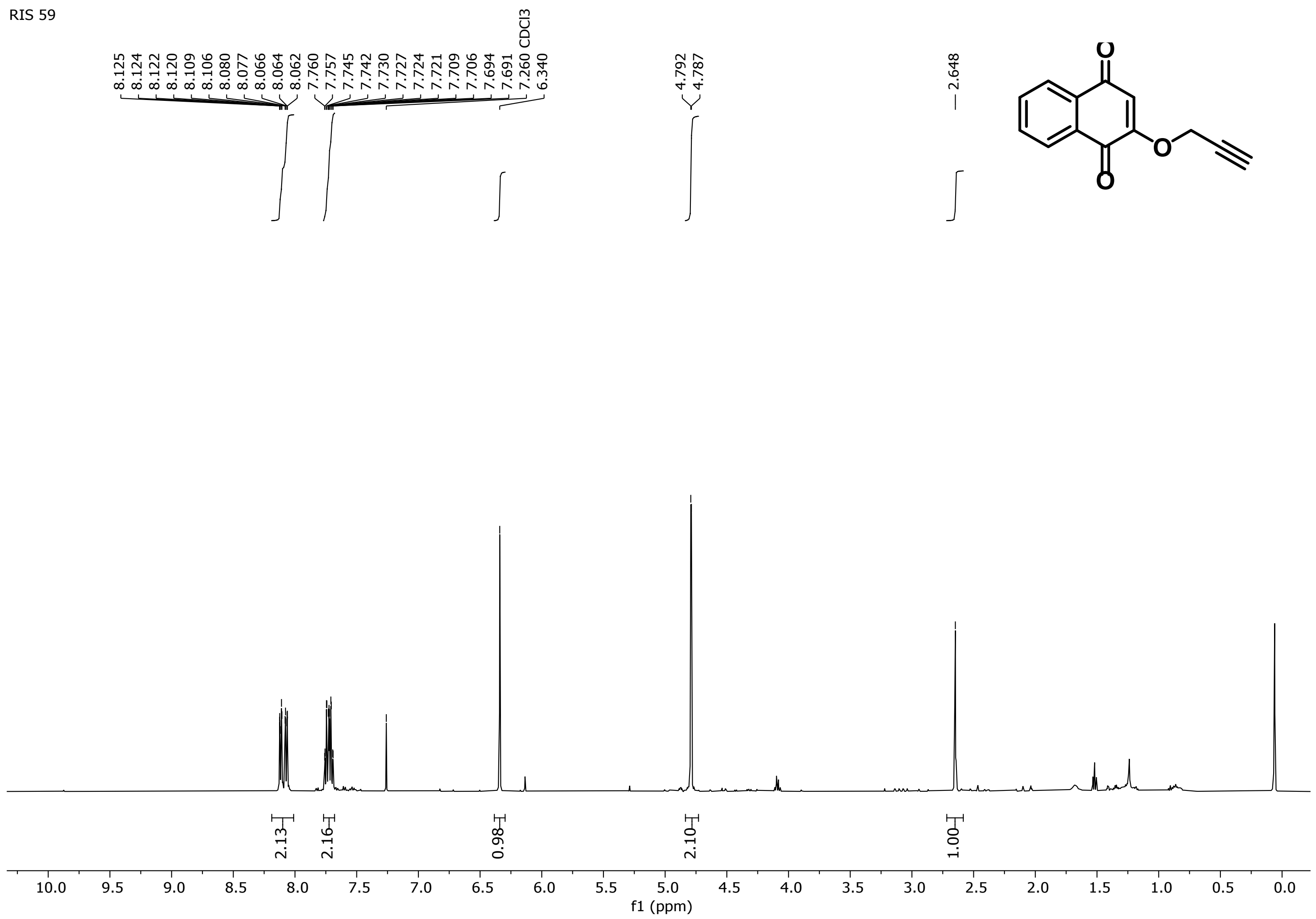


**Figure S23.** $^{1}$H NMR spectrum of **3g** (500 MHz, $CDCl_3$).

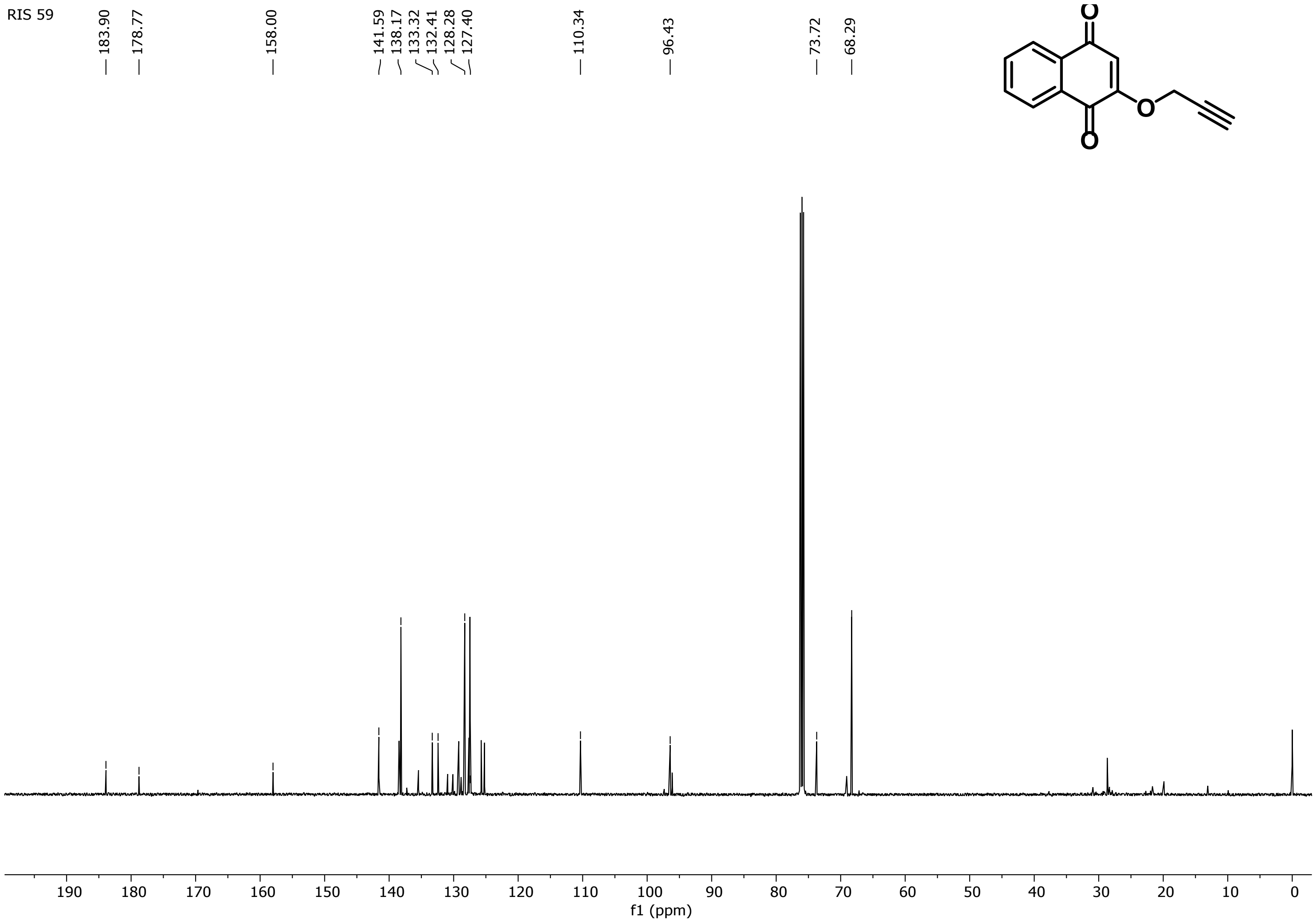


**Figure S24.** $^{13}$C NMR spectrum of **3g** (125 MHz, $CDCl_3$).

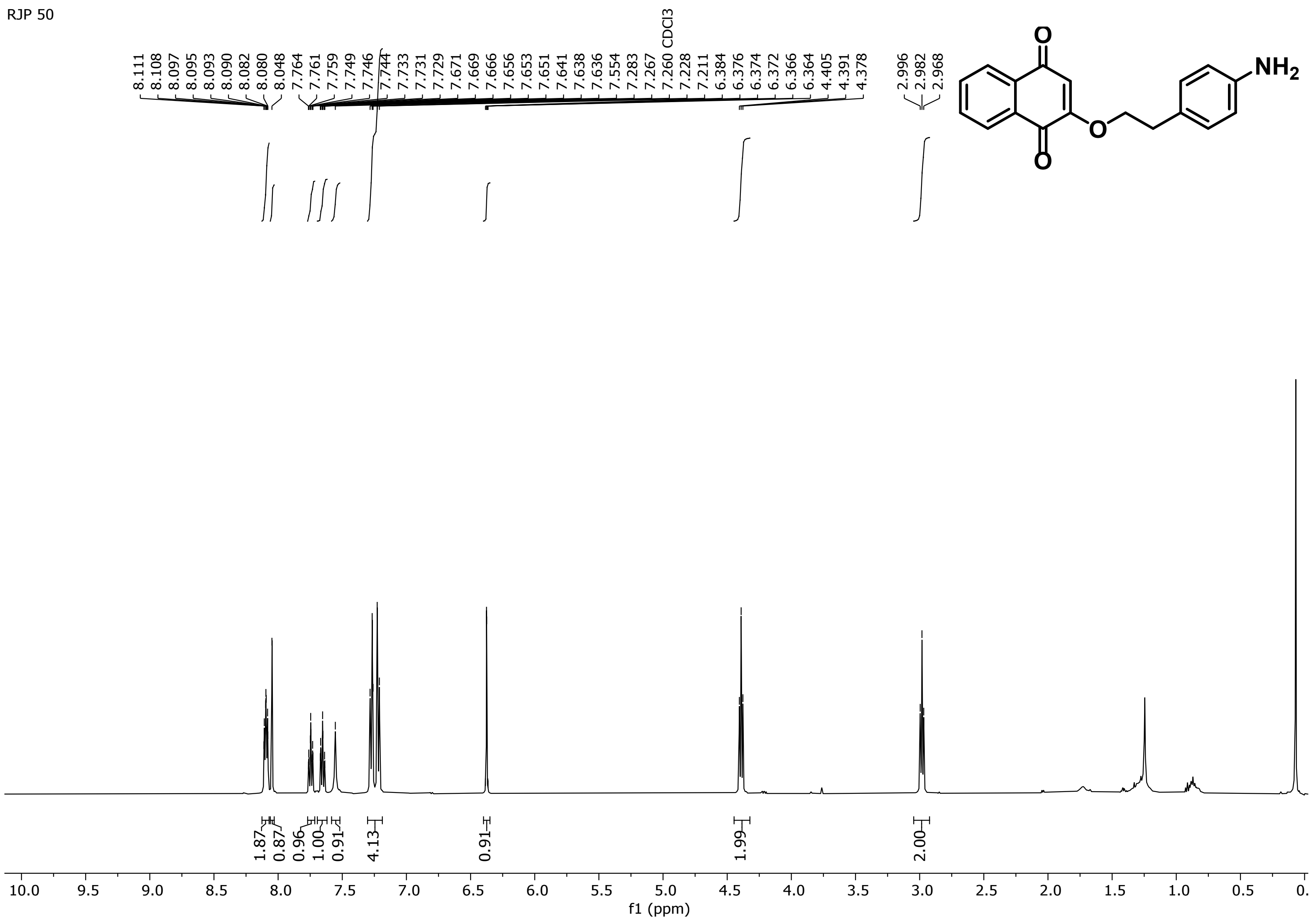


**Figure S25.** $^{1}$H NMR spectrum of **3h** (500 MHz, $CDCl_3$).

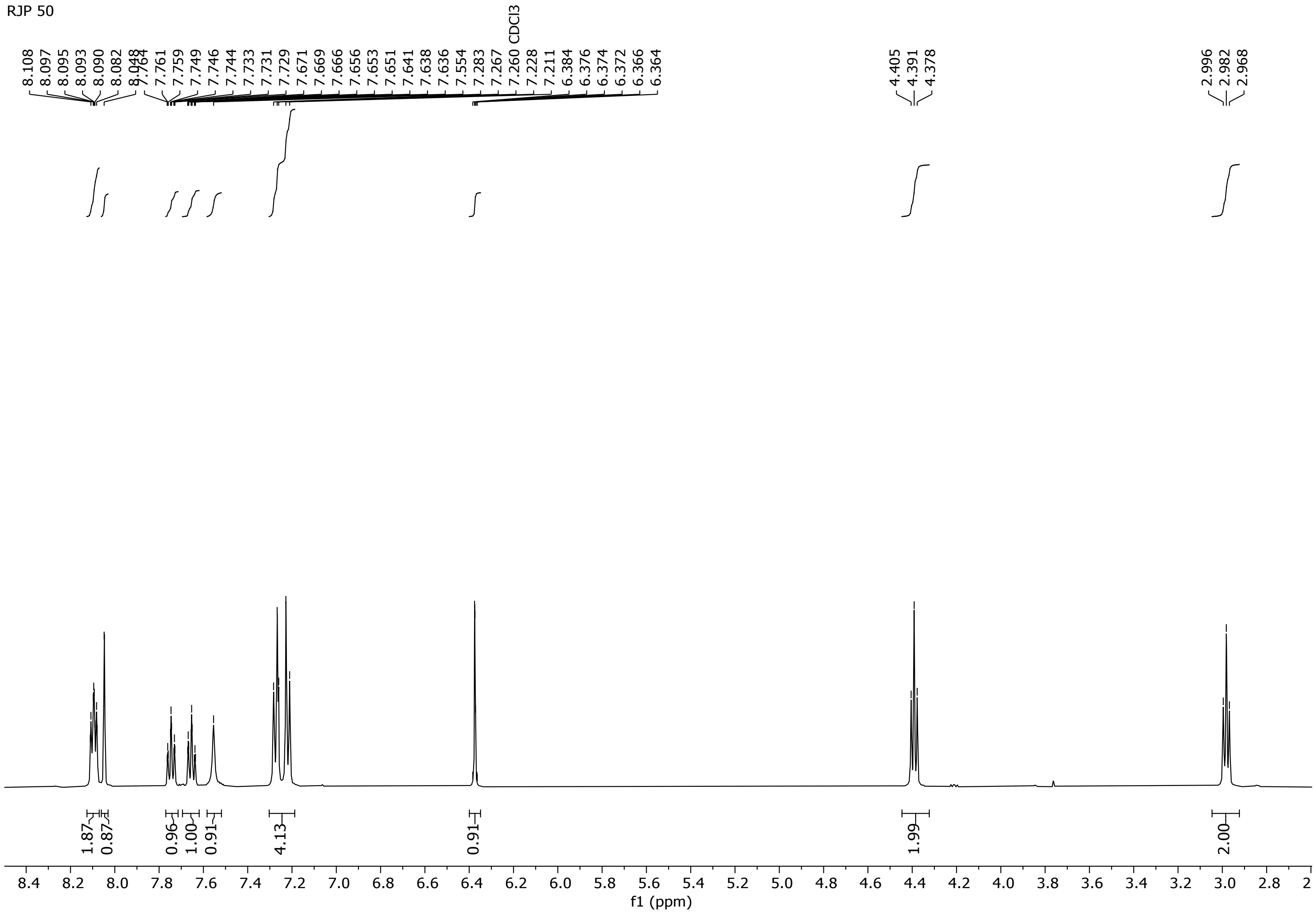


**Figure S26.** $^{1}H$ NMR spectrum expansion of **3h** (500 MHz, $CDCl_3$).

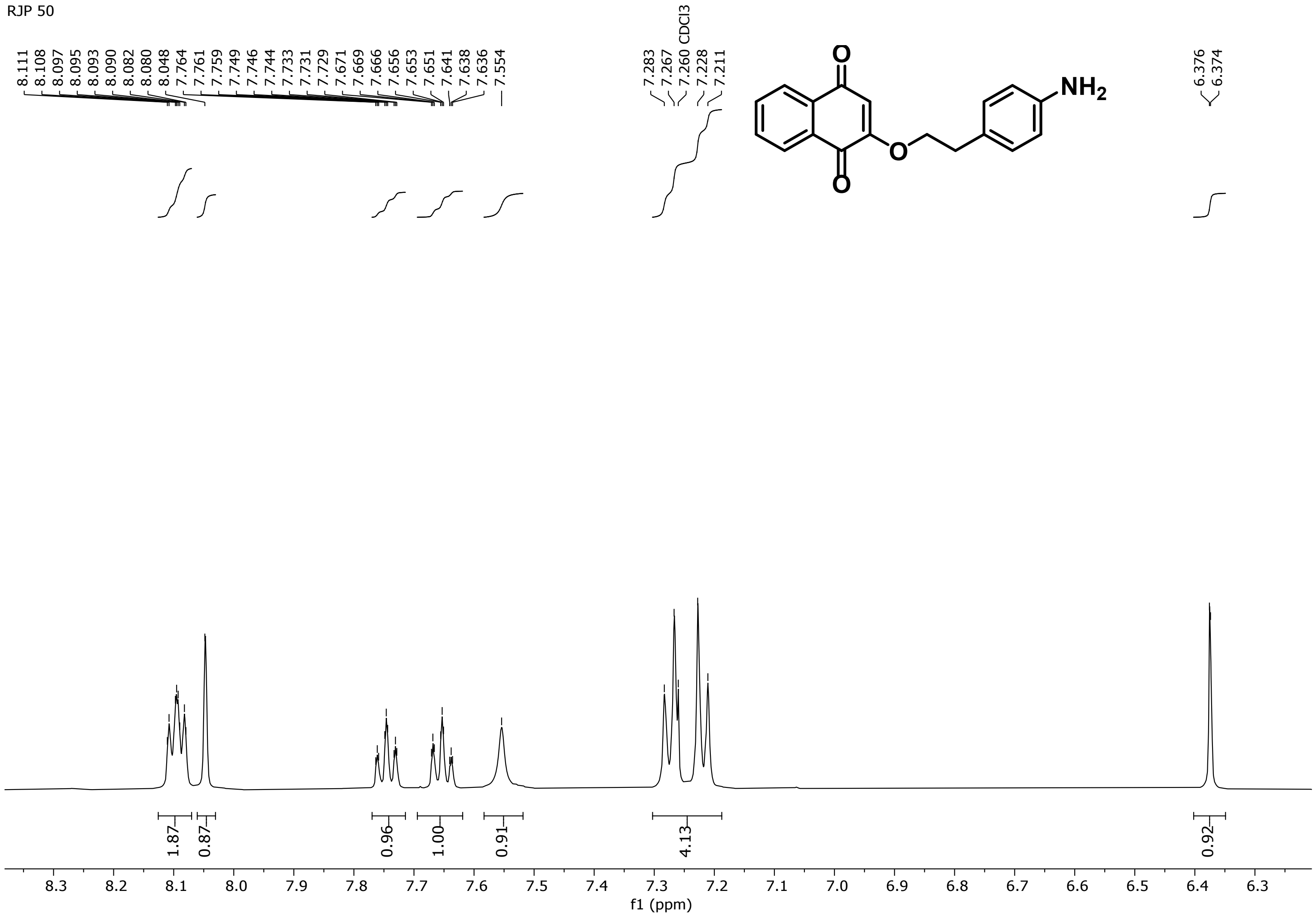


**Figure S27.** $^1$H NMR spectrum expansion of **3h** (500 MHz, $CDCl_3$).

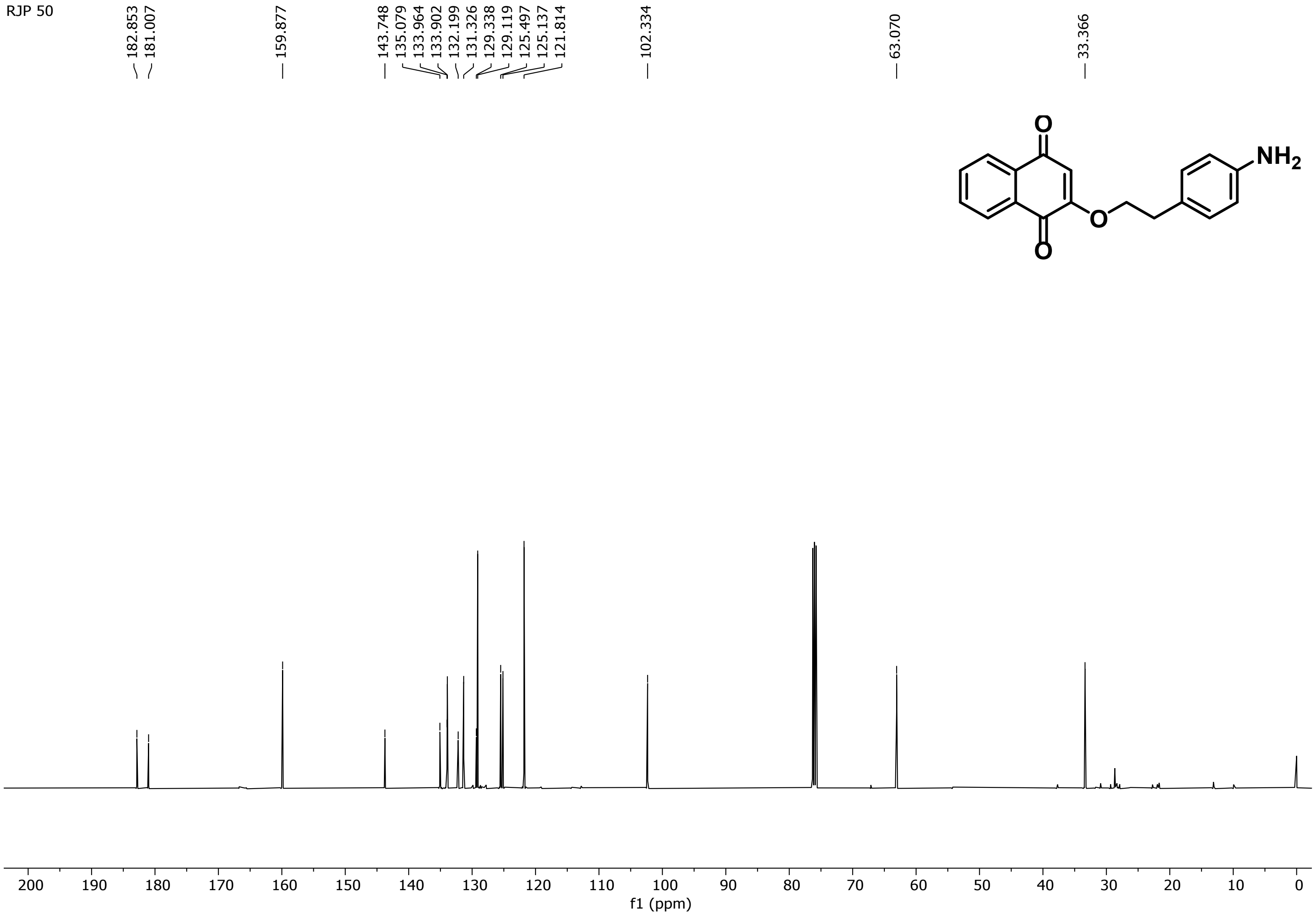


**Figure S28.** $^{13}$C NMR spectrum of **3h** (125 MHz, $CDCl_3$).

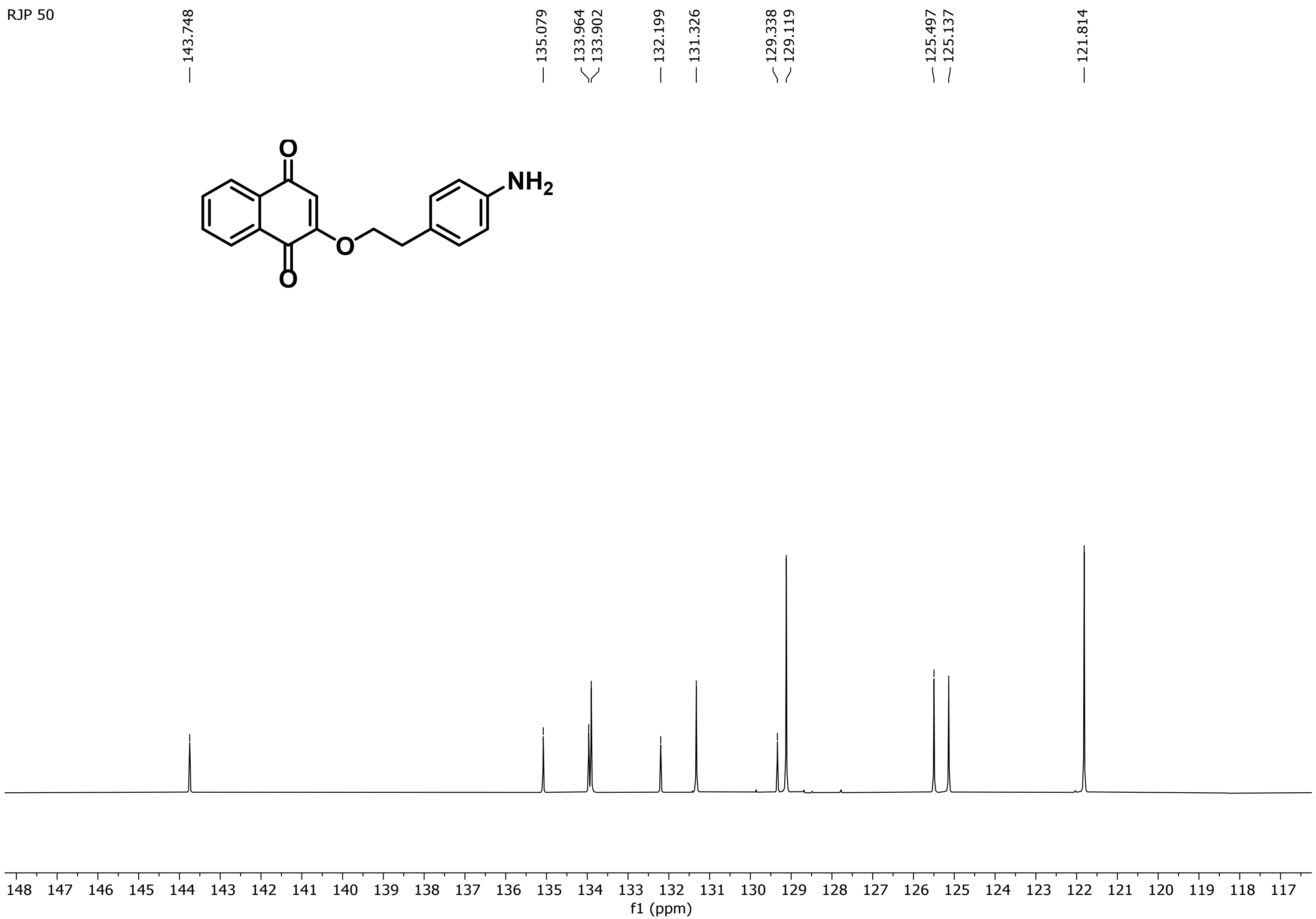


**Figure S29.** $^{13}$C NMR spectrum expansion of **3h** (125 MHz, $CDCl_3$).

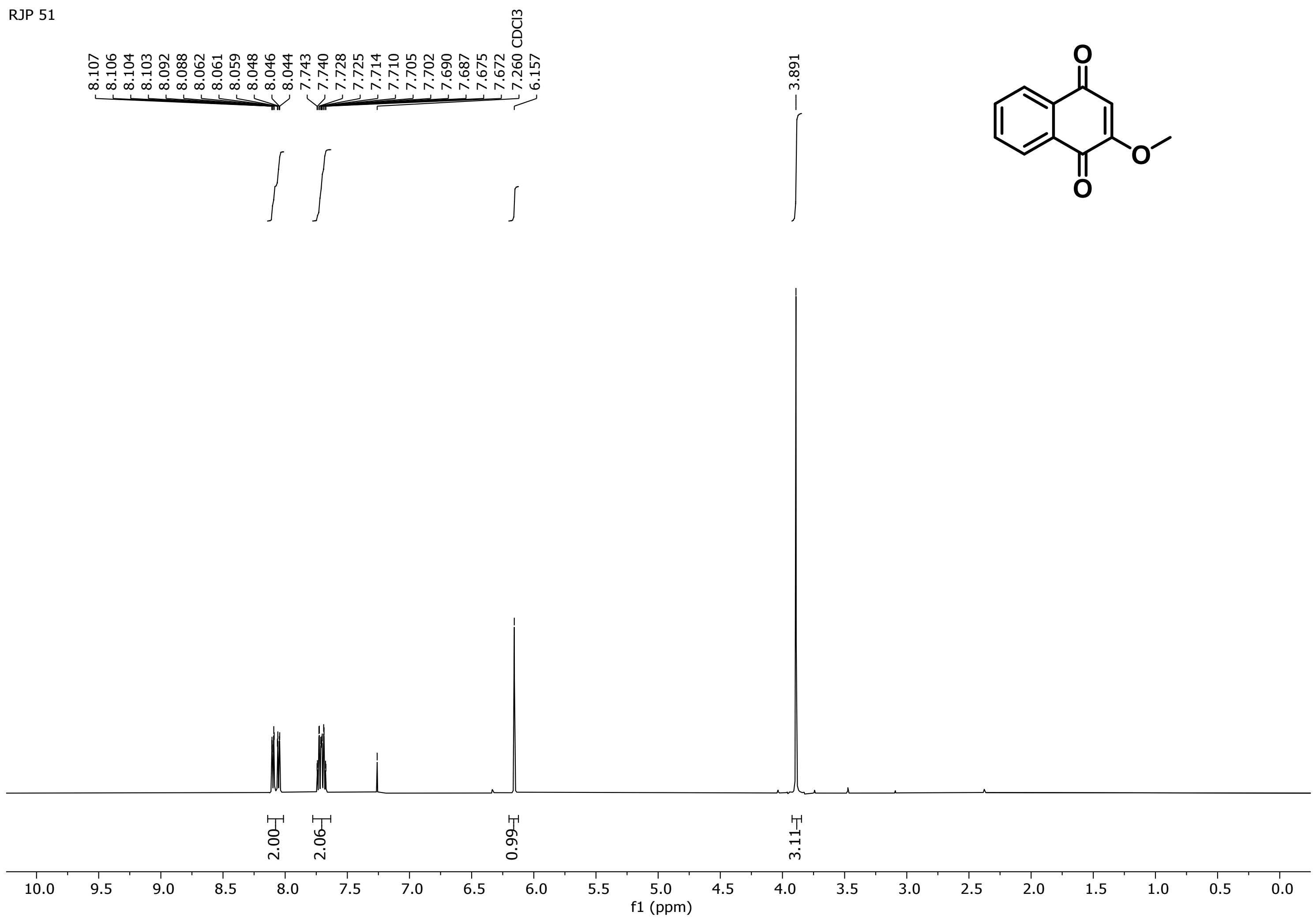


**Figure S30.** $^1$H NMR spectrum of **3i** (500 MHz, $CDCl_3$).

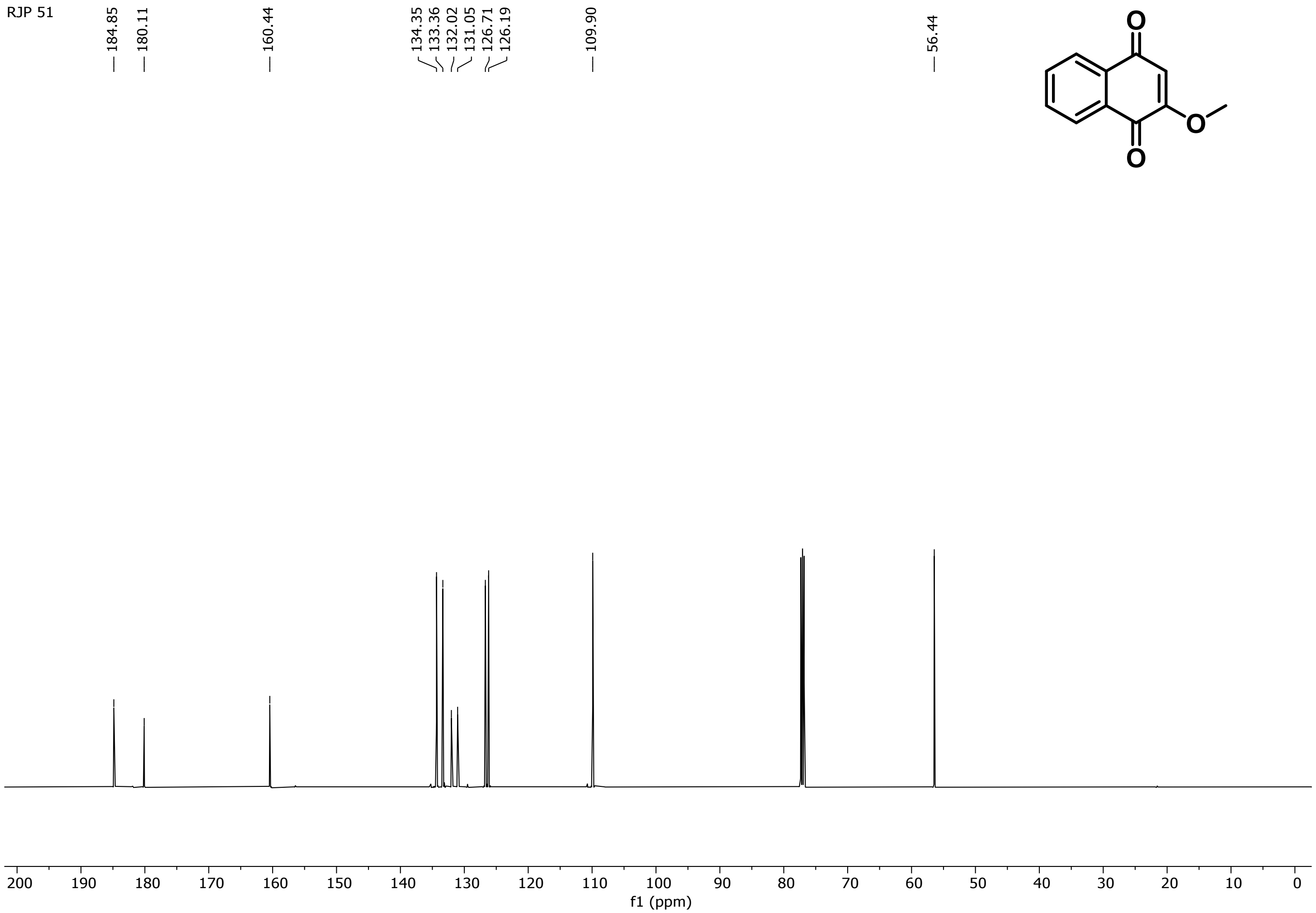


**Figure S31.** $^{13}$C NMR spectrum of **3i** (125 MHz, $CDCl_3$).

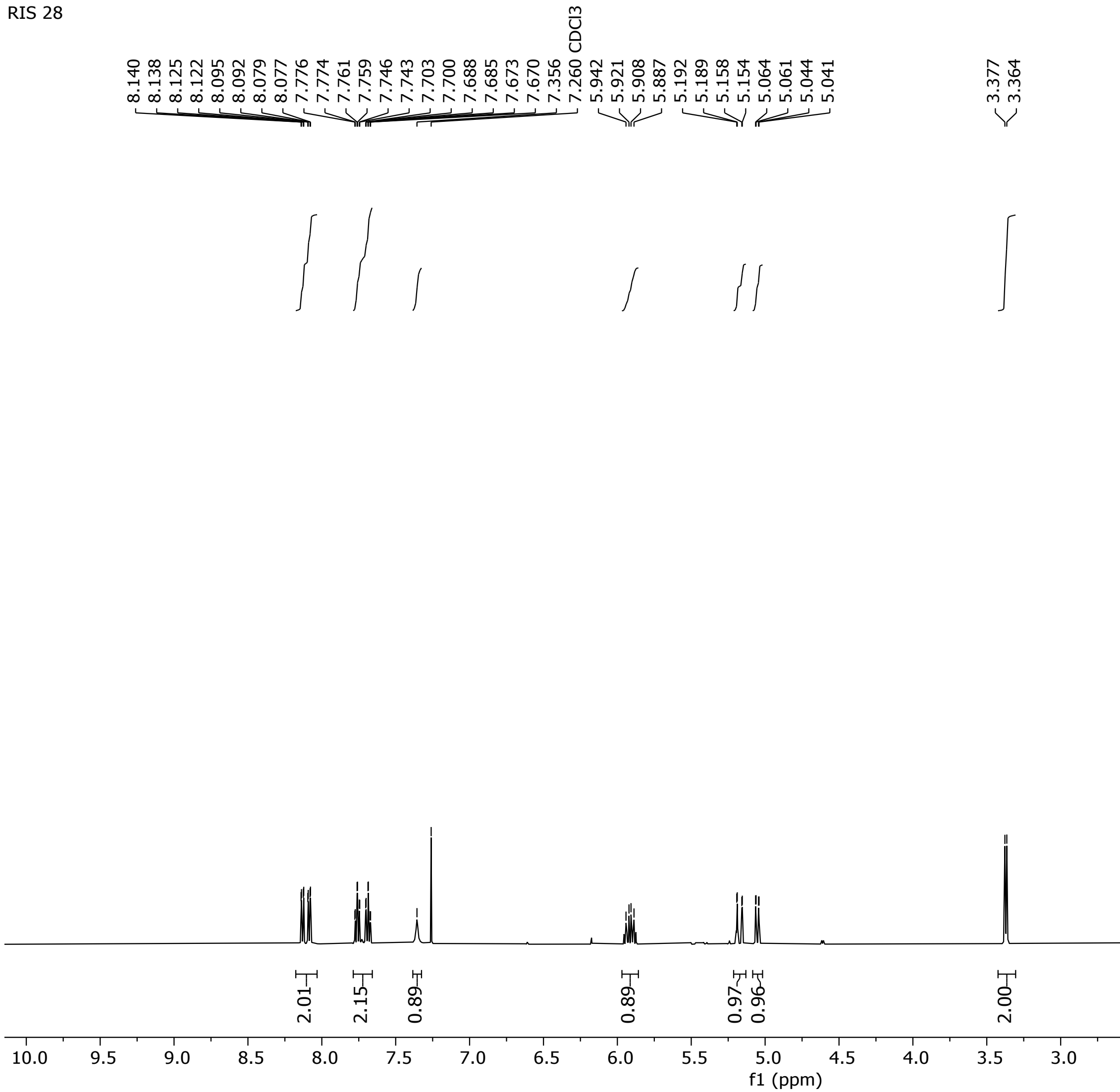


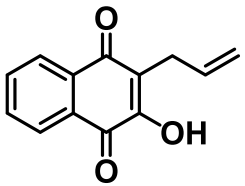


**Figure S32.** $^{1}$H NMR spectrum of **3i** (500 MHz, $CDCl_3$).

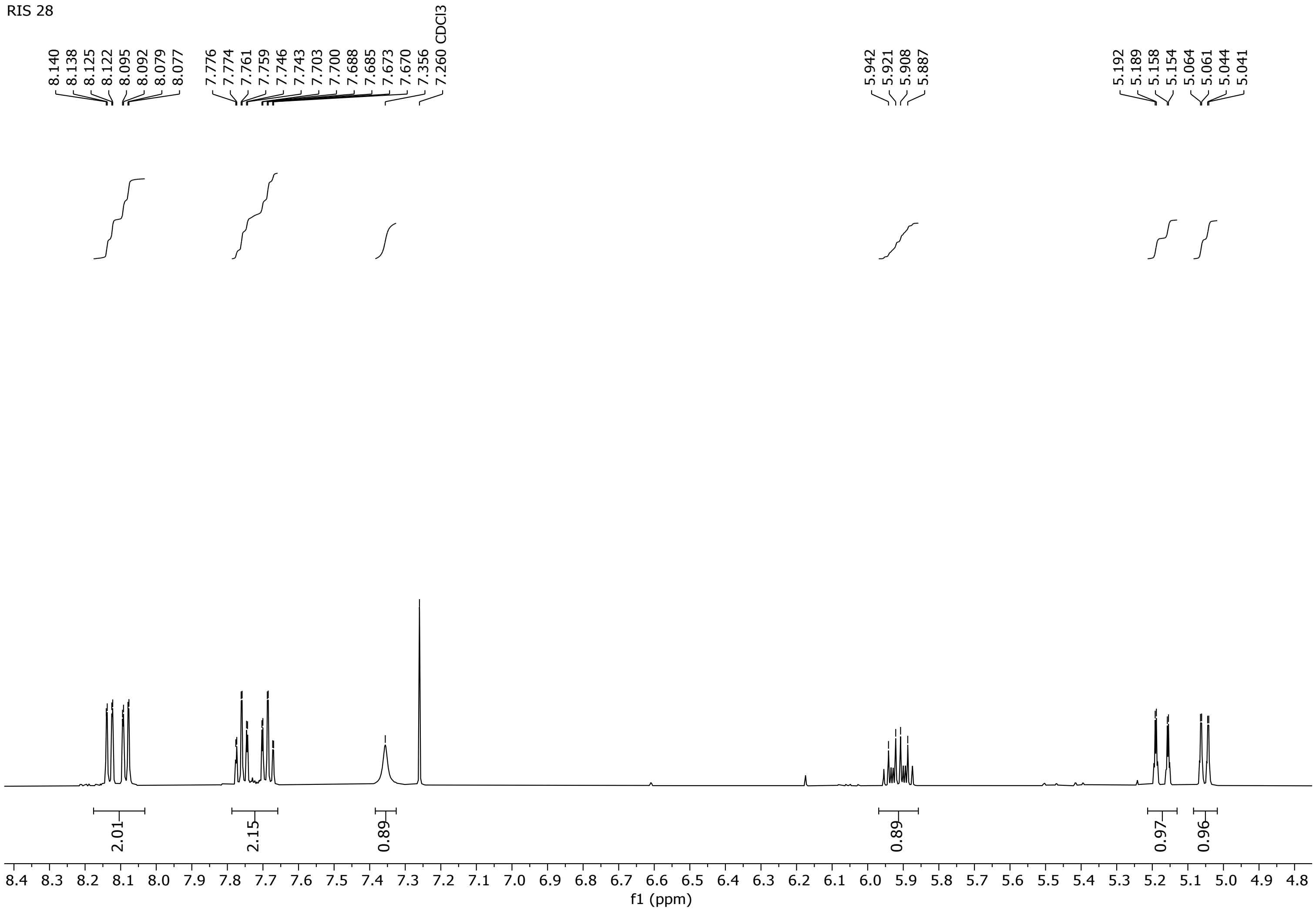


**Figure S33.** $^{1}$H NMR spectrum expansion of **3i** (500 MHz, $CDCl_3$).
